# Bone Metastasis: Molecular Mechanisms, Clinical Management, and Therapeutic Targets

**DOI:** 10.1002/mco2.70604

**Published:** 2026-02-05

**Authors:** Jingyuan Wen, Binghua Li, Shengjia Wang, Yongzhong Yao, Zhao Huang, Decai Yu

**Affiliations:** ^1^ Division of Hepatobiliary and Transplantation Surgery Department of General Surgery Nanjing Drum Tower Hospital, The Affiliated Hospital of Nanjing University Medical School Nanjing China; ^2^ Department of Breast Surgery Department of General Surgery Nanjing Drum Tower Hospital, the Affiliated Hospital of Nanjing University Medical School Nanjing China; ^3^ Department of Urology Fudan University Shanghai Cancer Center Shanghai China; ^4^ Division of Hepato‐Pancreato‐Biliary Surgery Tongji Hospital, Tongji Medical College, Huazhong University of Science and Technology Wuhan China; ^5^ Clinical Medical Research Center for Hepatic Surgery of Hubei Province Wuhan China; ^6^ Hubei Key Laboratory of Hepato‐Pancreato‐Biliary Diseases Wuhan China

**Keywords:** bone metastasis, bone microenvironment, bone niche, bone‐targeting therapy, cancer–bone crosstalk

## Abstract

Bone metastasis (BoMet) is a common complication in various cancers. Approximately 20–30% of patients with cancer develop BoMet, which is most frequently associated with solid tumors, such as breast, prostate, and lung cancers. BoMet can lead to skeletal‐related events such as fractures, bone pain, and hypercalcemia, negatively affecting the patient's quality of life and markedly shortening overall survival. The development of BoMet is a complex, multistep process driven by dynamic interactions between tumor cells and the bone microenvironment. The bone microenvironment provides a supportive niche for disseminated tumor cells, where intricate signaling networks and stromal interactions regulate the initiation, dormancy, reactivation, and progression of BoMet. Although current bone‐targeted therapies can reduce the incidence of these complications, the clinical outcomes for patients with BoMet remain poor. Therefore, elucidating the molecular mechanisms governing these interactions is essential for identifying new therapeutic strategies. This review systematically explores the molecular drivers of BoMet progression, dynamic interactions within the metastatic niche, available preclinical models, established treatment modalities, and emerging therapeutic approaches. As fundamental research continues to advance toward clinical translation, the outlook for patients with BoMet is expected to improve significantly.

## Graphical abstract

This figure emphasizes current understanding of the regulatory networks, approach of diagnose, available preclinical models and clinical management of bone metastasis. to guide future therapeutic development. A deep understanding of these aspects enables the prevention of bone metastasis and the implementation of effective therapeutic strategies.

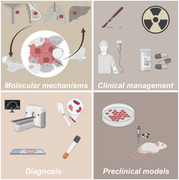

## 1 Introduction

Cancer is a leading causes of mortality globally [1]. Despite advances in managing primary tumors, most patients eventually develop metastases, leading to fatal complications [2]. Bone metastasis (BoMet) commonly occurs in breast cancer (BRCA), prostate cancer (PCa), and lung cancer, with BRCA and PCa accounting for over 80% of cases [3, 4]. Approximately 10% of patients with early‐stage PCa develop BoMet, whereas up to 80% of patients diagnosed at advanced stages present with metastases that primarily involve the axial skeleton [5]. Patients with castration‐resistant prostate cancer (CRPC) and estrogen receptor‐positive (ER^+^), progesterone receptor‐positive or human epidermal growth factor receptor (EGFR) 2‐positive BRCA are at increased risk of BoMet [6, 7]. Non‐small cell lung cancer (NSCLC) represents over 80% of lung cancer cases and occurs in approximately 30–40% of patients [8]. Peripheral lung adenocarcinomas demonstrate higher predilection for femoral and rib metastases, whereas central adenocarcinomas are stronger association with humeral involvement. Compared with patients without BoMet, those with BoMet exhibit a significantly poorer prognosis, with a median overall survival (OS) of approximately 5 months and a 5‐year survival rate below 5%.

Pathologically, BoMet lesions are classified as osteolytic, characterized by excessive bone resorption (common in advanced BRCA), or osteoblastic, characterized by excessive bone formation (frequent in PCa). These lesions often coexist in the same patient or site [9]. The repercussions of BoMet are often catastrophic and include severe bone pain, fractures, hypercalcemia, spinal cord compression, restricted mobility, and diminished quality of life [10]. Although multimodal therapeutic strategies (surgery, radiation, systemic therapy, and targeted agents) are used to manage BoMet, the prognosis remains poor. In addition, emerging evidence indicates that BoMet may confer resistance to immune checkpoint blockade (ICB) in extraosseous sites, further complicating treatment [11]. Thus, once established, BoMet is largely incurable, shifting treatment from curative to palliative, further burdening patients.

The development and progression of BoMet depend on the adaptation of tumor cell to the bone microenvironment (BME), which involves remodeling the microenvironmental, immune modulation, metabolic reprogramming, and cellular interactions that promote metastatic colonization and therapeutic resistance. This review provides a comprehensive overview of the current understanding of the epidemiology, pathophysiology, diagnosis, and treatment of BoMet in patients with cancer. It includes an in‐depth discussion of advances over the past decade in elucidating the molecular mechanisms underlying BoMet development, with particular emphasis on current bone‐targeted therapies and emerging therapeutic targets. Finally, future perspectives in this field are presented in the context of the latest research progress.

## 2 Pathophysiology of BoMet

BoMet is a progressive process. We detailed the molecular mechanisms that mediate each step and explained how cells in the BME, such as osteoclasts, osteoblasts, immune cells, and adipocytes, regulate the progression of BoMet through various interactions.

### 2.1 The Multistep Process of BoMet

**FIGURE 1:**
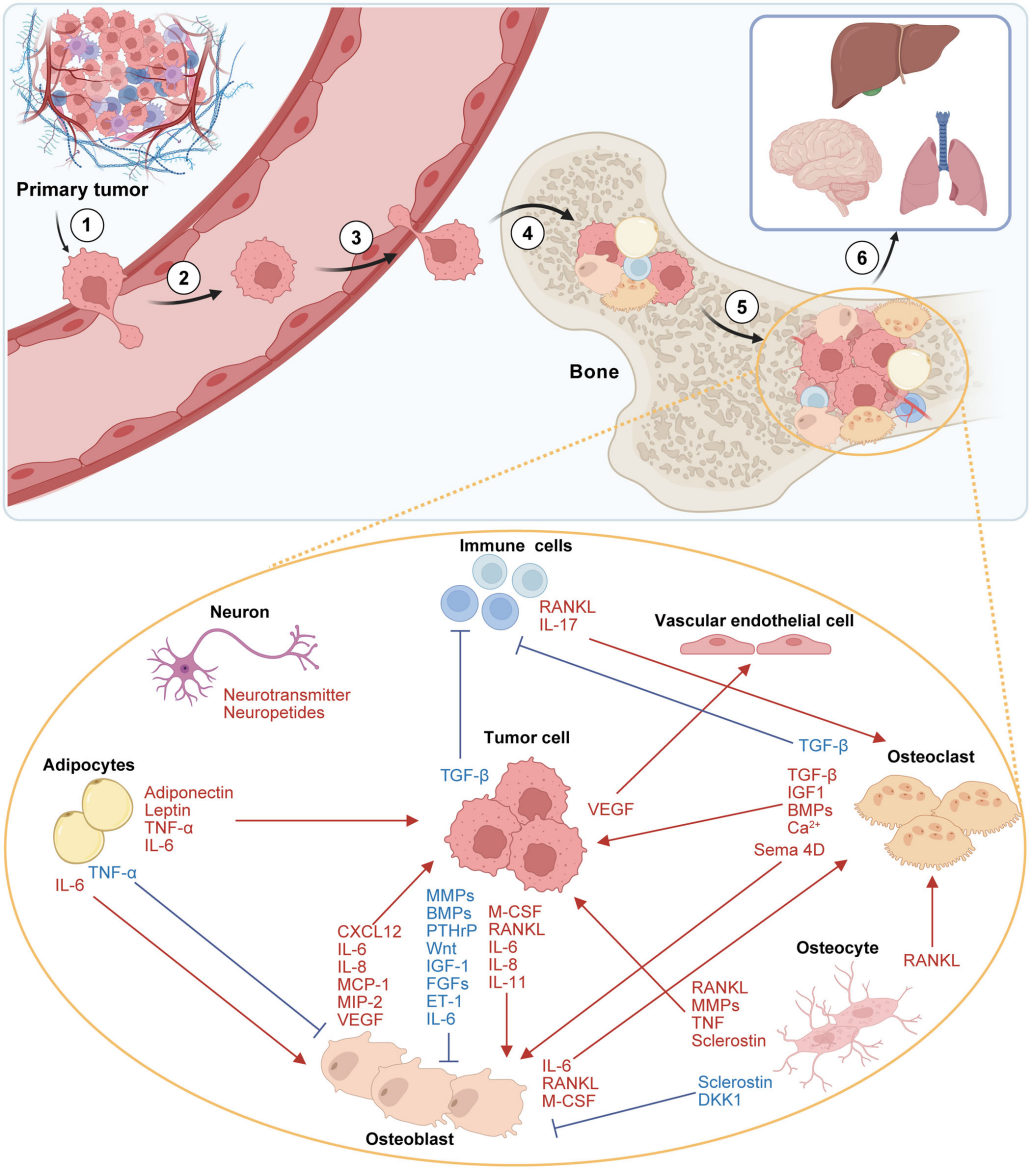
The multistep cascade of cancer bone metastasis and the metastatic bone microenvironment. The process of cancer metastasis from the primary site to bone is as follows: (1) cancer cells undergo malignant transformation, and the microenvironment enhances their motility and induces angiogenesis, increasing the likelihood that cancer cells will metastasize; (2) cancer cells spread through the blood vessel as CTCs; (3) CTCs extravasate and reach the bone metastasis site; (4) cancer cells enter the bone cavity, adhere to bone matrix cells, and deposit in the bone as DTCs; (5) through interactions with various bone matrix cells, DTCs either remain dormant or directly begin colonization and form micrometastatic foci, eventually thriving and expanding into large metastatic lesions within the bone; (6) cancer cells disseminate from established bone metastases to seed secondary tumors in other organs. Schematic illustration of crosstalk among various cells within the bone microenvironment. The bone marrow contains various cell types, including osteoblasts, osteoclasts, osteocytes, nerve cells, adipocytes, and so on. Cancer cell invasion disrupts bone homeostasis by regulating the secretion of multiple inflammatory factors and proteins that affect the differentiation and activity of bone cells, such as osteoblasts, osteoclasts, and immune cells. Conversely, bone‐resident cells influence cancer cell proliferation and metastasis through growth factors, cytokines, and chemokines. BMPs, bone morphogenetic proteins; CXCL, C–X–C motif chemokine ligand; DKK1, Dickkopf Wnt signaling pathway inhibitor 1; ET‐1, endothelin‐1; FGF, fibroblast growth factors; IGF, insulin‐like growth factor; IL, interleukin; MCP‐1, monocyte chemotactic protein‐1; MIP‐2, macrophage inflammatory protein 2; MMPs, matrix metalloproteinases; PTHrP, parathyroid hormone‐related protein; RANKL, receptor activator of nuclear factor kappa‐B ligand; Sema 4D, semaphorin 4D; TGF‐β, transforming growth factor‐β; TNF‐α, tumor necrosis factor α; VEGF, vascular endothelial growth factor. Created with BioRender.com.

Cancer metastasis is organ‐specific, with Paget's “seed and soil” doctrine describing metastatic cancer cells as “seeds” and the metastatic niche as the “soil” [12]. The reciprocal interaction between “seeds” and “soil” determines the organ‐specific spread of cancer [13]. Initially, cancer cells gain motility, invade tissues, and infiltrate blood vessels to access the bloodstream via the direct or lymphatic systems (Figure 1). Most tumors exhibit common prometastatic traits, including a dynamic epithelial–mesenchymal transition (EMT) program, which is crucial for detachment from the primary tumor [14]. Numerous tumor cell‐derived growth factors, including transforming growth factor‐β (TGF‐β), fibroblast growth factors (FGFs), and platelet‐derived growth factor (PDGF), activate the Wnt/β‐catenin and phosphoinositide 3‐kinase (PI3K)/protein kinase B (AKT)/mechanistic target of rapamycin (mTOR) signaling pathways and consequently enhance tumor cell proliferation, EMT, migration, and invasion [15]. However, since tumor cells often express epithelial markers, such as epithelial cell adhesion molecules and cytokeratin, EMT is not a necessary condition for determining premetastatic tumor dissemination [16, 17]. The tumor microenvironment (TME), comprising a network of the extracellular matrix (ECM), basement membrane, and vasculature, presents physical barriers that are degraded by matrix metalloproteinases (MMPs), thereby facilitating cancer cell intravasation [18, 19]. In addition, interactions between cancer cells and resident cells in the TME may give rise to a premetastatic niche in distant organs, subsequently promoting the homing of cancer cells [20].

Following extravasation into circulation, circulating tumor cells (CTCs) disseminate to distant organs and infiltrate the bone marrow during the early stages of primary tumor progression [21]. Prior to BoMet diagnosis, individual CTCs can be identified in the blood via “liquid biopsy.” This approach enhances risk stratification, improves prediction of cancer recurrence, and provides insights into the transition from chemotherapy sensitivity to resistance [22]. Even before cancer cells arrive in the bone, bioactive factors from primary tumors prepare the BME, facilitating cancer cell colonization [23]. Bone, particularly in axial regions like the spine and ribs, is highly vascularized. The trabecular bone in these areas has a rich blood supply and slow hemodynamics, which facilitate the adhesion and colonization of tumor cells on the endosteum. This process promoting BoMet. The successful colonization of disseminated tumor cells (DTCs) in the BME relies heavily on cell adhesion mechanisms [24]. Cancer cells frequently express integrin αvβ3, which mediates adhesion to various ECM components [7]. Similarly, various intrinsic factors within DTCs or their surrounding microenvironment, including growth arrest‐specific protein 6, TGF‐β2, and bone morphogenetic protein (BMP) 7, are essential for modulating the dormancy and reactivation of metastatic PCa cells in bone [25, 26, 27]. In fact, most colonized DTCs do not proliferate continuously on the bone surface where they are anchored but remain in a dormant state [28]. Dormant cancer cells evade CD8^+^ T‐cell‐mediated attacks by establishing an immunosuppressive environment through coordinated local hypoxia. These dormant cancer cells exhibit resistance to most radiotherapies and chemotherapies targeting proliferating cancer cells [29, 30]. Notably, DTCs can persist in a dormant state within the bone marrow for years, even after surgical removal of the primary tumor, contributing to the later recurrence of advanced BoMet [31]. Significant differences in the gene expression profiles between DTCs and primary cancer cells suggest that DTCs originate from specific tumor subclones that undergo subsequent genetic and epigenetic modifications [32]. For example, a subset of BRCA CTCs and DTCs exhibit altered ER and HER2 expression profiles compared with the primary tumor [33]. Colonization of DTCs in the bone is a highly complex and rate‐limiting step in the metastasis cascade, with only 0.01% of DTCs successfully colonizing and proliferating in distant organs [34]. The survival, dormancy, or colonization of cancer cells in bone is heavily influenced by the surrounding microenvironment (Figure 1) [35].

Beyond acting as a reservoir for dormant DTCs, the bone marrow also facilitates the recirculation of DTCs to other organs [21]. In a cohort of 367 female patients with BoMet, 228 patients developed extraosseous metastases. Consistent with these clinical observations, mouse models of BoMet also exhibited an increased tumor burden in distant organs [36]. The liver was the predominant site of secondary metastasis (51.4%), followed by the lungs (30.3%) and brain (13.8%) [37]. Although the mechanisms governing metastasis from bone to other organs are not fully defined, it is evident that tumor cells exposed to BME acquire enhanced capabilities for secondary dissemination. Cancer stem cells have a high propensity for bone tropism. Correspondingly, CTCs originating from BoMet display enhanced stem‐like attributes and clonogenic potential compared with those originating from primary tumors, thereby functioning as potent seeds for secondary dissemination [37]. Although cancer cells with stem‐like characteristics are often quiescent, they possess a remarkable capacity for long‐term persistence and inherent therapy resistance [37]. Following the establishment of BoMet, the enhancer of zeste 2 polycomb repressive complex 2 subunit (EZH2) within the metastatic niche enhances tumor cell aggressiveness. This epigenetic reprogramming endows tumor cells with stem‐like properties, thereby facilitating their spread [36]. Emerging evidence indicates that BME promotes the release of HER2^+^ BRCA CTCs. The acquisition of HER2 expression within the bone niche is critical for seeding BRCA cells from bone to other organs [38]. Immune modulation contributes to secondary metastases. For instance, apoptotic bodies derived from osteoclasts suppress naïve CD8^+^ T cell function via Siglec‐15, thereby promoting multi‐organ metastasis in advanced cancer with BoMet [39].

### 2.2 BMEs

The BME comprises osteoclasts, osteoblasts, osteocytes, immune cells, adipocytes, fibroblasts, endothelial cells, and their precursors, as well as the ECM [22]. These cellular and structural components interact to regulate bone remodeling, hematopoiesis, and BME homeostasis. Following colonization, cancer cell survival and clonal selection depend on BME support mechanisms, including niche hijacking, angiogenesis, stromal reprogramming, immune modulation, and evasion [40]. In this section, we discuss the primary cells that regulate BME homeostasis and their impact on metastatic cells (Figure 1).

#### 2.2.1 Osteoblasts

Mesenchymal stem cells (MSCs), the precursors of osteoblasts, originate from the mesoderm [41]. Their differentiation into osteoblasts is governed by a variety of intracellular and extracellular signaling molecules, including parathyroid hormone (PTH), Wnt, and Osterix [42]. Osteoblasts are responsible for producing organic bone ECM and differentiating into bone‐lining cells or osteocytes embedded in a mineralized matrix [41]. Cancer cells preferentially colonize trabecular bone regions in the metaphysis, which are characterized by a high abundance of osteoblasts [43]. Bodenstine et al. demonstrated that compared with the injection of BRCA cells alone, coinjection of BRCA cells and osteoblasts into the femur significantly increased tumor size in mice [44]. The C–X–C motif chemokine ligand 12 (CXCL12)/C–X–C motif chemokine receptor 4 (CXCR4) axis, involving CXCL12 from osteoblasts and CXCR4 from cancer cells, plays a crucial role in anchoring DTC BRCA cells to bone marrow [45]. Inhibiting the CXCL12–CXCR4 axis reactivates and mobilizes dormant cancer cells into the circulation [46]. In the presence of cancer cells, the levels of osteoblast‐secreted cytokines such as interleukin (IL)‐6, IL‐8, monocyte chemotactic protein‐1, macrophage inflammatory protein 2, and vascular endothelial growth factor (VEGF) increase [47]. These cytokines actively promote cancer cell invasion and metastasis, emphasizing the critical role of osteoblasts in cancer cell homing, colonization, and progression within the BME.

#### 2.2.2 Osteoclasts

Multinucleated osteoclasts derived from the monocyte–macrophage lineage of hematopoietic stem cells (HSCs) are key mediators of bone matrix resorption. The differentiation of these cells is primarily driven by macrophage colony‐stimulating factor (M‐CSF) and receptor activator of nuclear factor kappa B (NF‐κB) ligand (RANKL) [48]. Bioactive lipids facilitate osteoclast differentiation by activating tartrate‐resistant acid phosphatase (TRAP) and promoting multinucleation and bone matrix resorption [49]. In contrast, osteoprotegerin (OPG) produced by MSCs and osteoblasts inhibits osteoclast differentiation. In addition to their resorptive function, osteoclasts influence osteoblast development and activity through secreted factors, such as semaphorin 4D [50]. Additionally, TGF‐β and insulin‐like growth factor 1 (IGF‐1) are key regulators of both osteoblast and osteoclast functions [51]. Cancer cells upregulate osteoclastogenesis‐promoting factors, thereby accelerating bone resorption. This process releases growth factors that enhance cancer cell proliferation, migration, and colonization. For example, BRCA‐derived IL‐11 promotes osteoclast differentiation, and PTH‐related protein (PTHrP) secreted by tumor cells increase RANKL expression in osteoblasts [52]. Excessive bone resorption releases factors such as calcium, TGF‐β and IGF from the bone matrix, which fuel cancer growth and thus establish a vicious cycle between tumor proliferation and bone destruction [52]. Furthermore, in established BoMet, osteoclast‐derived osteopontin (OPN) enters the systemic circulation and the extraosseous TME. OPN impairs T‐cell recruitment and the differentiation of CD8^+^ TCF1^+^ progenitor cells, leading to reduced efficacy of ICB therapy [11].

BoMet is classified into two main types: osteolytic and osteoblastic. Osteolytic BoMet is characterized by localized bone destruction mediated by excessive osteoclast activity, appearing radiographically as “punched‐out” lesions. In contrast, osteoblastic BoMet is defined by increased osteoblast activity, resulting in bone sclerosis. These pathological forms often coexist at BoMet sites, forming “mixed” lesions that exhibit both bone resorption and sclerosis [53]. The BoMet BME is characterized by enhanced osteoclast‐mediated bone resorption, suppressed osteoblast‐driven bone formation (in osteolytic lesions), increased angiogenesis, and immunosuppression [52]. Abnormal cellular and molecular interactions during BoMet progression disrupt the balance between bone resorption and formation.

#### 2.2.3 Osteocytes

Osteocytes, which constitute approximately 90% of all the bone cells in the adult skeleton, are embedded within the bone matrix and serve as mechanosensors that regulate bone formation and resorption [54, 55]. Osteocytes derived from osteoblasts produce sclerostin (SOST) and Dickkopf‐related protein 1, which inhibit MSC‐derived osteoblast generation and suppress bone formation [56]. They are also the primary sources of RANKL in the skeleton, thereby controlling osteoclast growth and differentiation [57]. Osteocytes secrete various growth factors and cytokines such as RANKL, MMPs, tumor necrosis factor (TNF), and SOST, which play crucial roles in the migration, proliferation, and malignant progression of cancer cells [42]. Recent studies have demonstrated a novel mechanism by which mitochondria from osteocytes can be transferred to cancer cells via tunneling nanotubes. This intercellular mitochondrial transfer, mediated by Miro1 and MFn2, elevates cytoplasmic mitochondrial DNA levels in cancer cells, subsequently activating the cGAS–STING signaling pathway. This activation enhances tumor immunogenicity and promotes antitumor immune responses [58]. However, direct evidence linking osteocytes to BoMet regulation requires further investigation.

#### 2.2.4 Bone Marrow Adipocytes

In adults, approximately 15% of the marrow is occupied by adipocytes, a proportion that increases to 60% by the age of 65 years [59]. Derived from bone marrow MSCs, bone marrow adipocytes (BMAs) differentiation is promoted by peroxisome proliferator‐activated receptor (PPAR) activation and inhibited by β‐catenin via Wnt signaling, which favors osteogenesis over adipogenesis [60, 61]. BMAs are metabolically active and contribute significantly to BoMet by regulating adjacent cells. For example, a high‐fat diet increases BMA abundance, facilitating PCa BoMet in mice [62]. BMAs serve as triglyceride reservoirs, modulate fatty acid (FA) metabolism, and influence nearby cells through autocrine, paracrine, and endocrine pathways [63, 64]. Specifically, BMAs secrete IL‐6, which stimulates osteoblasts to produce RANKL, thereby promoting osteoclastogenesis. This process facilitates EMT in cancer cells and the growth of other BME cells [65]. In addition, BMAs activate NF‐κB signaling in osteoblasts by secreting TNF‐α, thereby inhibiting their differentiation [66]. Moreover, TNF‐α induces the expression and secretion of RANKL in BMAs, thereby stimulating the differentiation and activation of osteoclasts [67]. Previous studies have shown that BRCA cells exhibit preferential and targeted migration toward BMAs [68]. Leptin, an adipocyte‐derived hormone, promotes bone resorption and creates a microenvironment that supports the growth of cancer cells in the bone marrow [69]. Elevated levels of leptin and IL‐1β enhance the migration of BRCA toward the conditioned medium of human bone tissue and promote their colonization of the bone marrow adipose tissue niche [68]. A high‐fat diet induces the expansion of the BMA population, specifically accelerating cancer progression in bone [62]. Corroborating this, cancer cells cultured with BMA‐conditioned media showed increased proliferation and migration, which was attributed to the upregulation of FA transport proteins, such as cluster of differentiation 36 (CD36) and FA‐binding protein 4 (FABP4), leading to intracellular lipid accumulation [62]. The direct coculture of cancer cells with BMAs reduces the lipid content in BMAs and downregulates the expression of genes such as FABP4, adiponectin, and resistin [62, 70]. Collectively, these findings underscore the importance of BMAs in regulating BoMet.

#### 2.2.5 Immune Cells

Lymphocytes, including T, B, and natural killer (NK) cells, originate from HSCs and lymphoid progenitors in the bone marrow [71]. Cancer BoMet often develops in an immunosuppressive microenvironment where T cells are exhausted or inactive [72]. For example, TGF‐β released by osteoclasts suppresses T‐cell‐mediated antitumor responses [73]. Regulatory T cells (Tregs) derived from CD4^+^ T cells, which are elevated in the bone marrow of PCa patients with BoMet, suppress immune responses and promote osteoclast differentiation via CXCR4/CXCL12 signaling [74, 75]. In addition, FOXP3^+^ Tregs, which are the key sources of RANKL, facilitate osteoclast differentiation, cancer migration, and BoMet progression [76]. Similarly, tumor‐specific Th17 cells produce RANKL, which activates osteoclasts and drives osteolytic bone disease [75]. Furthermore, RANKL/RANK signaling activation modulates the NF‐κB pathway to suppress B cell apoptosis and enhance B cell survival/proliferation. This creates an immune‐protective niche that facilitates immune evasion by cancer cells, ultimately inhibiting tumor cell apoptosis and promoting BoMet [77]. NK cells directly recognize cancer cells via antigen‐specific receptors and induce cancer cell apoptosis through cytotoxic granule‐mediated exocytosis or receptor‒ligand interactions [78]. In the BME, cancer cells expressing Core 2 β‐1,6‐N‐acetylglucosaminyltransferase inhibit ligand‒receptor‐mediated immune responses, thereby preventing apoptosis [79]. In BRCA, inhibiting TGF‐β signaling enhances NK cell antitumor activity and prevents BoMet [73]. Studies on NK cell‐depleted mice have demonstrated that administering PTHrP‐neutralizing antibodies, bisphosphonates, activin inhibitors, and VEGF antibodies inhibits lung cancer BoMet [73]. However, the precise mechanisms underlying the role of NK cells in BoMet remain unclear.

Macrophages are mononuclear myeloid cells that polarize into the proinflammatory M1 or anti‐inflammatory M2 phenotypes [80]. M1 macrophages are tumor‐suppressive and secrete proinflammatory cytokines that activate cytotoxic T and NK cells [81]. In contrast, M2 macrophages, also known as tumor‐associated macrophages (TAMs), secrete cytokines that suppress CD4^+^ and CD8^+^ T cell activity [82]. Previous studies demonstrated an increase in the number of CD206^+^ M2 macrophages in PCa with BoMet lesions [83]. Macrophage depletion through genetic targeting or pharmacological approaches inhibits tumor growth in the bone [84]. BRCA cells expressing CCL2 recruit C–C motif receptor 2 (CCR2)^+^ macrophages and preosteoclasts to promote bone colonization [85]. In addition, M‐CSF functions as a chemotactic factor and governs the proliferation and differentiation of osteoclasts as well as the macrophage‐mediated cancer BoMet [86]. Under pathological conditions, immature myeloid cells fail to differentiate and accumulate as immunosuppressive myeloid‐derived suppressor cells (MDSCs) [87]. The injection of MDSCs induces recurrence in mice with BoMet but has no effect on healthy mice [88, 89]. Furthermore, MDSCs differentiate into osteoclasts, thereby promoting bone destruction [75]. MDSCs promote the production of IL‐17 in the bone, which enhances osteoclastogenesis via RANKL signaling [90]. MDSCs also express CCR2 and CCL2, which enhances osteoclast activity [91]. Furthermore, MDSCs produce VEGFA, which upregulates E‐selectin to enhance tumor cell adhesion, thereby facilitating the homing and proliferation of CTCs. Moreover, MDSCs secrete MMP9 to potentiate VEGF activity, promoting angiogenesis as well as tumor cell extravasation and migration [15]. Dendritic cells (DCs), which are derived from multipotent HSCs, are potent antigen‐presenting cells capable of inducing cytotoxic T lymphocytes [92]. Circulating DCs display a pronounced tendency to migrate to the bone marrow, where VCAM‐1 and E‐selectin promote DCs retention within the BME [93]. Increased numbers of plasmacytoid DCs were observed in the bone marrow of mice implanted with BRCA cells [94]. The PDCA‐1‐induced deficiency of plasmacytoid DCs substantially inhibited BoMet, underscoring the potential of plasmacytoid DC‐targeted therapies for BoMet treatment. Additionally, plasmacytoid DCs recruit Tregs and MDSCs to facilitate cancer progression and metastasis, rather than protecting against it [95]. Although the functions of various immune cells within the TME are well characterized, the role of immune cells within the BME remains poorly understood and warrants further investigation.

#### 2.2.6 Nerve Cells

Recent studies have highlighted the role of neural cells in the BME, which contains sympathetic, sensory, and glutamatergic nerves [96]. Psychological stress activates the sympathetic nervous system, altering the bone marrow stroma and facilitating BRCA cell colonization [97, 98]. Sympathetic nerves in the cortex release norepinephrine, which binds to β2‐adrenergic receptors on osteoblasts, leading to the upregulation of RANKL and promoting osteoclast formation and bone resorption [98]. Thus, the nervous system plays a critical role in bone homeostasis.

## 3 Molecular Mechanisms of Regulating Bone Metastases

In this section, we explain the complexity of the regulatory mechanisms involved in the BoMet process from multiple perspectives, including signaling pathways, immunomodulatory molecules, and lipid metabolites.

### 3.1 Pathway

#### 3.1.1 Wnt Signaling Pathway

**FIGURE 2:**
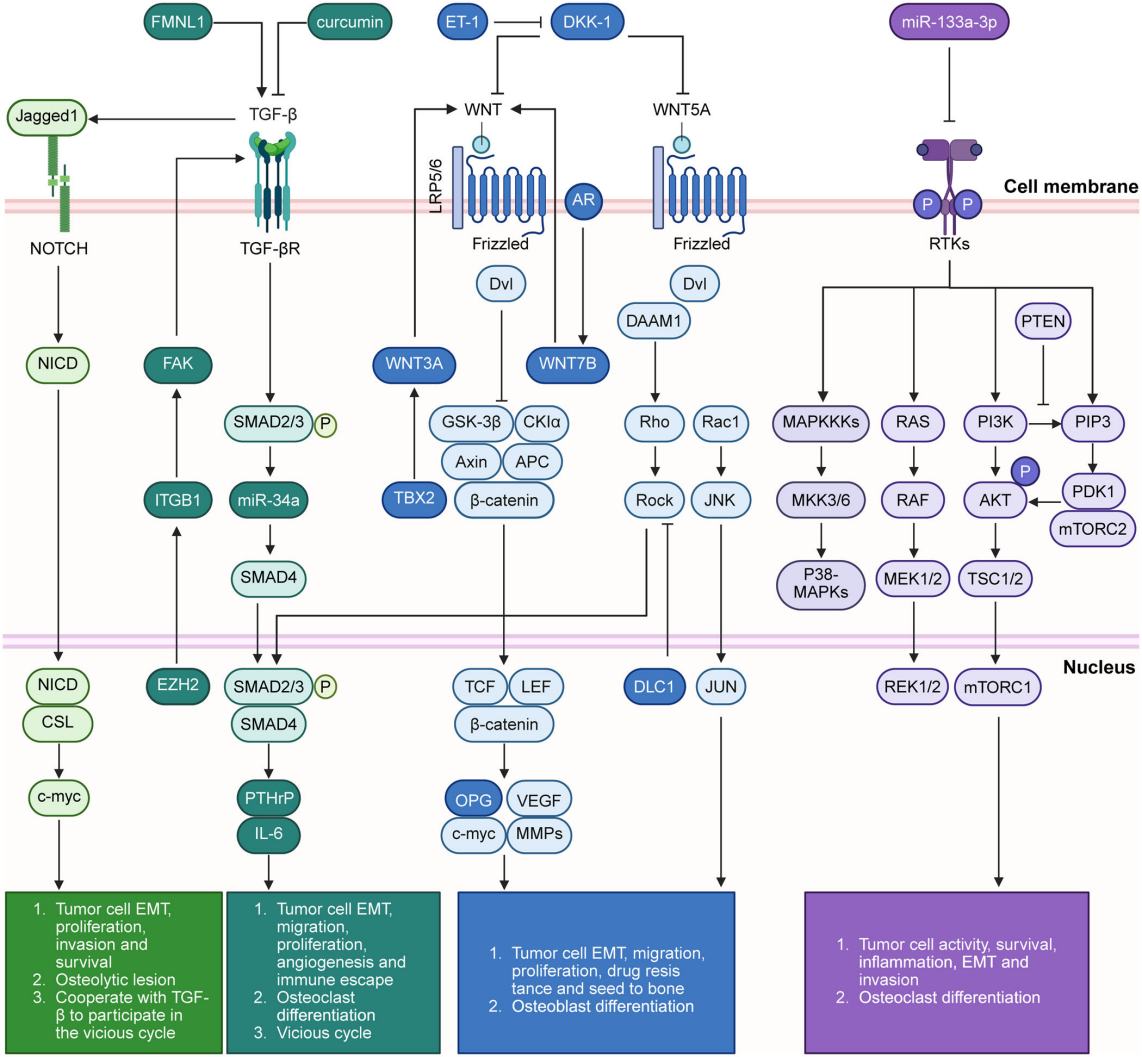
Signaling pathways involved in bone metastasis. Secreted proteins mediate intercellular signaling between tumor cells and the bone microenvironment. Signaling cascades from various pathways regulate both tumor cell behavior and the surrounding bone niche. Dvl, Dishevelled; EMT, epithelial–mesenchymal transition; EZH2, enhancer of zeste 2 polycomb repressive complex 2 subunit; FAK, focal adhesion kinase; FMNL1, formin‐like protein 1; ITGB1, integrin β1; JUNK, c‐Jun N‐terminal kinase; NICD, Notch intracellular domain; OPG, osteoprotegerin; PDK1, pyruvate dehydrogenase kinase 1; PTEN, phosphatase and tensin homolog; RTKs, receptor tyrosine kinase. Created with BioRender.com.

Vertebrates express 19 Wnt ligands. Representative canonical ligands include Wnt3, Wnt3a, Wnt7a, Wnt8, and Wnt10, whereas Wnt4, Wnt5a, Wnt5b, and Wnt11 are classified as noncanonical [99]. In the canonical Wnt pathway, a destruction complex composed of Axin, CK1α, adenomatous Polyposis Coli (APC), and GSK3β facilitates β‐catenin phosphorylation, targeting it for ubiquitination and proteasomal degradation (Figure 2). Pathway activation is initiated by ligand binding to Frizzled and LRP5/6 coreceptors. This event induces LRP5/6 phosphorylation, leading to the recruitment of Axin and Dishevelled (Dvl) to the plasma membrane. Consequently, Frizzled is phosphorylated and activated, while Axin is dephosphorylated and degraded. Membrane‐recruited Dvl inhibits GSK3 activity, thus preventing β‐catenin degradation. Stabilized β‐catenin accumulates in the cytoplasm, translocates to the nucleus, and associates with T‐cell factor (TCF)/Lymphoid enhancer factor (LEF) transcription factors to regulate target gene expression [100]. The noncanonical Wnt signaling pathways primarily include the planar cell polarity (PCP) and the Wnt/calcium pathway. The PCP pathway transmits signals through Dvl upon Wnt ligand–receptor engagement, leading to the activation of the small GTPases Rho and Rac. Rho activation is initiated by Wnt‐induced Dvl–Daam1 complex formation, in which Daam1 recruits the RhoGEF WGEF to activate Rho. This, in turn, stimulates Rho‐associated kinase (ROCK) activity, ultimately driving actin cytoskeletal remodeling. The Rac pathway originates with Dvl activation, resulting in the sequential activation of Rac GTPase and the subsequent stimulation of the c‐Jun N‐terminal kinase (JNK) [100].

Canonical Wnt ligands promote MSC commitment to the osteoblastic lineage. Sustained pathway activity in mature osteoblasts orchestrates terminal differentiation into osteocytes and modulates osteoblast apoptosis. Furthermore, Wnt/β‐catenin signaling in osteoblasts and osteocytes upregulates OPG, thereby suppressing osteoclast activity and bone resorption [99]. Although the role of Wnt in bone development and homeostasis is well established, its function in carcinogenesis is complex and remains incompletely understood [101]. Activation of Wnt signaling may induce EMT in various cancers, primarily through the Wnt/β‐catenin pathway, which activates the transcription factors zinc finger E‐box‐binding homeobox (ZEB) and Snail and suppresses E‐cadherin expression [99]. In PCa, the transcription factor T‐box transcription factor 2 regulates WNT3A expression, thereby enhancing metastatic dissemination, including cellular invasion and BoMet [102]. The noncanonical Wnt ligand Wnt5a may modulate BoMet expression in PCa via the Wnt‐PCP pathway, facilitating metastatic seeding at skeletal sites [99]. However, osteoblast‐derived Wnt5a mediates the activation of noncanonical Wnt signaling, which suppresses the canonical Wnt pathway and consequently initiates or maintains cellular dormancy in PCa cells. Wnt7b, which is highly expressed in human PCa tumors associated with osteoblastic lesions and is regulated by the androgen receptor (AR), mediates osteoblast differentiation through direct cell‐cell interactions [103]. In addition, the activation of Wnt signaling induces osteolytic metastasis, which is characterized by the upregulated expression of bone sialoprotein, OPN, CXCR‐4, and PTHrP [99]. As a member of the Dickkopf family, Dickkopf Wnt signaling pathway inhibitor 1 (DKK1) binds to LRP5/6 and blocks Wnt ligand–receptor interaction, thereby suppressing the canonical Wnt/β‐catenin signaling pathway [104]. Zhuang et al. proposed that tumor‐secreted DKK1 is a potential serum biomarker for metastatic organotropism in BRCA [105]. DKK1 suppresses lung cancer cell metastasis via antagonizing the noncanonical Wnt/PCP–RAC1–JNK axis, while also enhancing BoMet by modulating osteoblastic canonical Wnt signaling to inhibit OPG secretion [105]. Finally, cancer cell‐secreted endothelin‐1 (ET‐1) in the BoMet niche coordinately modulates Wnt signaling by repressing DKK1 and promoting Wnt5a expression [99].

#### 3.1.2 TGF‐β Signaling Pathway

Upon TGF‐β ligand binding to its membrane receptors, the Type II receptor transphosphorylates the Type I receptor, enabling Smad recruitment and activation (Figure 2). Subsequently, phosphorylated Smad accumulates in the nucleus to govern transcriptional responses [106]. TGF‐β belongs to the polypeptide growth factor superfamily, which also comprises activins, inhibins, and BMPs. BMPs signal via specific Type I and Type II receptors to activate Smad1/5/8. Phosphorylated Smad1/5/8 forms heteromeric complexes with the common mediator Smad4 and translocates to the nucleus [106]. Beyond the canonical pathway, TGF‐β can also directly initiate non‐Smad signaling cascades. For instance, TβRI directly phosphorylates Shc, leading to subsequent Erk activation. Additionally, small GTPases (Ras, Rho, Rac, and Cdc42) mediate essential non‐Smad TGF‐β signaling responses.

TGF‐β exhibits a dual role in cancer, initially acting as a tumor suppressor in early stages but promoting malignancy once resistance to its antiproliferative effects develops [106]. Overall, TGF‐β orchestrates advanced malignant progression by coordinating EMT‐mediated cellular plasticity, invasive dissemination, angiogenic switching, and immune suppression [106]. TGF‐β is one of the most abundant cytokines embedded within the bone matrix. During osteolytic BoMet, excessive osteoclast activity and resultant elevated bone resorption liberate substantial amounts of matrix‐bound TGF‐β into the local microenvironment. This TGF‐β‐rich milieu activates signaling cascades in both tumor and stromal cells, prompting tumor cells to secrete pro‐osteoclastic factors (such as PTHrP, IL‐6 and, Jagged1) that further stimulate osteoclast differentiation and activation, thus establishing a self‐reinforcing vicious cycle [107]. EZH2‐mediated upregulation of ITGB1 initiates a signaling cascade through focal adhesion kinase (FAK) activation, leading to enhanced TGF‐β pathway activity and BRCA BoMet [108]. By targeting the ras homolog family (RHO)–ROCK pathway, DLC1 attenuates TGF‐β signaling by inhibiting SMAD3 phosphorylation, resulting in reduced PTHLH production and subsequent suppression of osteoclast maturation, ultimately blocking the vicious cycle of osteolytic metastasis in BRCA [109].

#### 3.1.3 Notch Signaling

In mammals, the Notch signaling pathway consists of four receptors (Notch1‐4) and five canonical membrane‐bound ligands (Jagged1, Jagged2, DLL1, DLL3, and DLL4). Ligand–receptor binding triggers sequential proteolytic cleavage of the receptor, first by ADAM metalloproteases and then by the γ‐secretase complex, resulting in the release of the Notch intracellular domain (NICD). NICD then translocates to the nucleus, assembles with the CSL transcription factor, and activates the transcription of target genes (Figure 2) [110].

Notch receptors are upregulated in various tumors and promote EMT, thereby enhancing metastatic dissemination [110]. Notch3 messenger ribonucleic acid (mRNA) expression is elevated in NSCLC tumor tissues from patients with BoMet [111]. The knockdown of Notch3 reduces the invasive and migratory capacities of NSCLC cells and suppresses TGF‐β‐induced expression of PTHrP and IL‐6 [111]. Notch3 promotes EMT and BoMet in NSCLC cells through ZEB‐1. In addition, tumor cell‐derived Jagged1 activates osteoclast function and stimulates osteoblasts to secrete protumorigenic factors, such as IL‐6, collectively promoting bone destruction and cancer growth. This osteolytic process releases bone matrix‐derived factors (e.g., TGF‐β), which further upregulate Jagged1 expression in cancer cells, establishing a self‐reinforcing vicious cycle. Notably, the 15D11 anti‐Jagged1 monoclonal antibody reduced BRCA BoMet in preclinical models, validating Notch pathway inhibition as a promising therapeutic approach [107].

#### 3.1.4 PI3K/AKT and MAPK Signaling

The core components of the PI3K/AKT pathway include Class I PI3K, AKT serine/threonine kinases, and downstream effectors. Class I PI3K functions as a heterodimer comprising a p110 catalytic subunit and a p85 regulatory subunit and is subdivided into Classes 1A and 1B based on regulatory subunit composition (Figure 2). Class IA PI3K is primarily activated by growth factor receptor tyrosine kinases, whereas Class IB responds to G protein‐coupled receptors. AKT, the central serine/threonine kinase downstream of PI3K, has three isoforms (AKT1–3) encoded by distinct genes. Upon extracellular ligand binding, membrane receptors recruit PI3K to the plasma membrane, where they phosphorylate PIP_2_ to generate PIP_3_. PIP_3_ subsequently serves as a secondary messenger that recruits AKT and pyruvate dehydrogenase kinase 1 (PDK1) to the membrane. PDK1 phosphorylates AKT at Thr308, and its subsequent phosphorylation at Ser473 by mTORC2 results in complete AKT activation [112]. The PI3K/AKT pathway promotes BoMet by directly enhancing tumor cell motility, invasiveness, and survival, as well as modulating tumor‐stromal crosstalk to remodel BME and disrupt physiological bone remodeling [113]. In PCa cells, miR‐133a‐3p suppresses PI3K/AKT signaling by directly targeting several growth factor receptors, including the EGFR, FGFR1, IGF1R, and mesenchymal‐epithelial transition factor (MET), thereby inhibiting PCa BoMet [114].

The MAPK signaling pathway comprises four major cascades: extracellular signal‐regulated kinase (ERK), p38, JNK, and ERK5. Activation of p38 MAPK is essential for the expression of multiple inflammatory cytokines and chemokines [115]. Notably, the p38 MAPK pathway, particularly the p38α isoform, plays a critical role in RANKL‐mediated osteoclast differentiation. Mitogen‐activated protein kinase 2 (MK2), a downstream effector of p38 MAPK, is essential for osteoclastogenesis. Targeted inhibition of p38 MAPK or MK2 in the stromal compartment of metastatic lesions reduces the production of stromal‐derived tumor‐promoting cytokines such as IL‐6, thereby suppressing metastatic tumor growth [116].

### 3.2 Immune System

#### 3.2.1 Complement System

The innate immune system recognizes tumor cells as dangerous nonself‐entities and mounts potent cytotoxic responses against them, a process enhanced by the synergistic action of tumor‐specific antibodies and the complement system [117]. The complement system is a central component of innate immunity and is primarily activated through three major pathways: (1) the classical pathway, triggered by antigen–antibody complexes binding to C1q, leading to the sequential activation of C1r and C1s and culminating in the cleavage of C4 and C2 to form the C3 convertase (C4b2a); (2) the lectin pathway, in which mannose‐binding lectin or ficolins directly recognize carbohydrate motifs on pathogen surfaces, triggering MBL‐associated serine proteases and sharing downstream events with the classical pathway; and (3) the alternative pathway, characterized by the spontaneous hydrolysis of C3 in plasma, its subsequent binding to factor B, and cleavage by factor D to generate the initial C3 convertase (C3bBb). All three pathways converge during the formation of C3 convertase, which cleaves C3 into C3a and C3b. C3b binds to C5 convertase, leading to C5 activation [117].

Although the role of the complement system in antitumor immunity is well established, emerging evidence indicates that it also regulates BoMet. Specifically, the G protein‐coupled receptor C5aR1 (CD88) is expressed in lung cancer cells, and the C5a/C5aR1 axis enhances their migratory and invasive abilities [118]. In vivo studies demonstrated that both C5aR1 knockdown and pharmacological blockade of C5aR1 using AOND21 effectively suppressed BoMet in lung cancer. This effect is attributed to the inhibition of C5aR1, which reduces the expression and secretion of the chemokine CXCL16 in tumor cells, thereby inhibiting osteoclast differentiation (Table 1) [118].

**TABLE 1:** Immune factor contributing to BoMet.

Factor	Receptor	Category	Tumor	Mechanism	References
C5a	C5aR1	Complement	Lung cancer	Tumor cell migration and invasion; Osteoclast differentiation	[118]
IL‐1β	IL‐1R1	Cytokines IL‐1 family	PCa, BRCA	Tumor cell EMT and actin cytoskeleton remodeling; MDSCs differentiation and immunosuppressive capacity; Angiogenesis	[119, 120, 121, 122]
IL‐4	IL‐4R	Cytokines IL‐2 family	BRCA	Macrophages M2 polarization	[123]
IL‐6	IL‐6R	Cytokines IL‐6 family	BRCA	Osteoclast differentiation	[124]
IL‐11	IL‐11Rα; GP130	Cytokines IL‐6 family	BRCA	Osteoclast differentiation	[124]
IL‐20	IL‐20RA; IL‐20RB	Cytokines IL‐10 family	Lung cancer	Tumor cell proliferation in BME	[125]
M‐CSF	CSF1R	Colony stimulating factor	Lung cancer	Tumor cell proliferation and invasion	[126]
G‐CSF	CSF3R	Colony stimulating factor	BRCA	Angiogenesis	[127]
IL8	CXCR1;CXCR2	Chemokine	—	Osteoblast maturation and activation	[128, 129]
CXCL5	CXCR2	Chemokine	PCa, BRCA	Tumor cell colonization and proliferation in BME	[130, 131]
CCL2	CCR2	Chemokine	PCa	Macrophage infiltration; tumor cell proliferation	[132]
CXCL12	CXCR4	Chemokine	PCa	Tumor cells home to specific BoMet niches	[103, 133]
CD137	CD137L	Tumor necrosis factor	BRCA	Osteoclast differentiation	[132, 134]

#### 3.2.2 Cytokines

Both tumor and inflammatory cells secrete proinflammatory cytokines that contribute to the formation of premetastatic niches, which in turn regulate the activation, proliferation, and migration of tumor cells within the BME [15].

Elevated IL‐1β expression in both PCa and BRCA promotes tumor cell metastasis to bone (Table 1). IL‐1β drives BRCA cell proliferation by inducing EMT and promoting osteolysis [119]. The PI3K/Rac axis mediates IL‐1β‐induced actin cytoskeleton remodeling in BRCA cells, thereby enhancing their invasiveness [120]. IL‐1β stimulates bone marrow cells to upregulate immunosuppressive gene expression, promoting the differentiation of granulocytes into MDSCs. Incubation with IL‐1β in vitro markedly enhances the expression of immunosuppressive markers on MDSCs and increases their capacity to inhibit T cell proliferation [121]. IL‐1β mediates its biological effects by binding to IL‐1R1. Anakinra, a recombinant IL‐1 receptor antagonist, directly suppresses tumor cell proliferation and angiogenesis within and beyond the BME [122].

BME‐associated macrophages exhibit high expression of the IL‐4 receptor (IL‐4R), promoting their differentiation toward the M2 phenotype. Macrophage‐specific conditional knockout of IL‐4R significantly reduces the incidence of BRCA BoMet [123].

IL‐6 and IL‐11 play pivotal roles in osteoclastogenesis within the BoMet niche. While IL‐6 promotes osteoclast formation in an osteoblast‐dependent manner, IL‐11 directly induces osteoclast differentiation. Elevated IL‐11 expression in BRCA is closely associated with an increased incidence of BoMet. Experimental models have shown that IL‐11 enhances tumor burden and osteolytic lesions by promoting RANKL‐independent osteoclastogenesis through the JAK1/signal transducer and activator of transcription 3 (STAT3) pathway, which upregulates c‐Myc, a key regulator of osteoclast differentiation [124].

The IL‐20 receptor subunit beta (IL‐20RB) forms heterodimeric complexes with either IL‐20RA or IL‐22 receptor (IL‐22R) to bind the IL‐20 subfamily cytokines (IL‐19, IL‐20, and IL‐24) [135]. Compared with normal lung tissues, IL‐20RB expression was significantly upregulated in lung tumors, with further elevation observed in bone‐tropic A549 sublines and clinical bone metastatic lesions [126]. Patients with elevated IL‐20RB expression have an increased risk of skeletal recurrence and poorer OS. Osteoclast‐derived IL‐9 binds to IL‐20RB in lung cancer cells, triggering the activation of the intracellular iJAK1–STAT3 pathway, which drives tumor cell proliferation within BME [126].

Colony‐stimulating factor 1 (CSF1) exerts its biological effects through its receptor (CSF1R), which is highly expressed in mononuclear phagocytes, osteoclasts, and certain cancer cell populations [136]. Lung cancer cells coexpress CSF1 and CSF1R, and elevated CSF1 expression significantly enhances the proliferative and invasive capacities of A549 cells in vitro. Intracardiac injection experiments demonstrated that CSF1 knockdown in A549 cells significantly reduced the BoMet burden, attenuated osteolytic lesions, and decreased Ki‐67 positivity [126].

BRCA cell‐derived granulocyte CSF (G‐CSF) directly remodels the vascular endothelium, independently of hematopoietic cells. Therapeutic blockade of G‐CSF receptors within the metastatic microenvironment suppresses pathological vascular remodeling, thereby alleviating BoMet (Table 1) [127].

#### 3.2.3 Chemokine

IL‐8 promotes osteoblast maturation and activation, leading to the subsequent release of bone matrix‐degrading factors, such as acid phosphatase and MMPs, and thereby exacerbating osteolytic destruction [128]. Furthermore, IL‐8 stimulates RANKL secretion and suppresses OPG production in bone marrow stem cells, resulting in an imbalanced RANKL/OPG ratio [129].

Previous studies have identified CXCL5 as a key mediator of metastatic colonization, as it promotes the proliferation and establishment of BRCA cells within the bone. The pharmacological inhibition of its receptor CXCR2 with specific antagonists effectively suppresses the proliferation of metastatic BRCA cells [130]. In bone marrow MSCs, MDA‐9, when stimulated with tumor cell‐derived PDGF‐arachidonic acid (AA), activates the Hippo signaling pathway to induce CXCL5 secretion, thereby establishing a microenvironment conducive to PCa BoMet [131].

CCL2, a tumor‐derived chemokine, enhances monocyte migration to inflammatory sites. Bone marrow endothelial cells promote the recruitment of PCa cells to osseous tissues by secreting high levels of CCL2. Mechanistically, the binding of CCL2 to CCR2 on macrophages induces their infiltration into tumor tissues, further promoting tumor cell proliferation [132].

Elevated CXCR4 expression was observed in osteolytic lesions of patients with BoMet [133]. CXCL12 expression is low in the diaphysis but significantly higher in the metaphysis of long bones (the primary anatomical site where most osteoblasts and PCa cells reside) [103]. Blockade of the CXCL12/CXCR4 axis with AMD3100 mobilized PCa cells from their homing niches without affecting cell viability. These findings suggest that the CXCL12/CXCR4 axis mediates the homing of PCa cells to specific niches in the bone marrow.

CD137, which is expressed on macrophages, binds to its ligand CD137L on tumor cells, triggering Fra1 upregulation. This process facilitates monocyte migration into the tumor stroma and promotes their differentiation into osteoclasts, thereby contributing to BRCA BoMet [132]. Notably, a novel F4/80‐targeted lipid nanoparticle (NP) encapsulating an anti‐CD137 blocking antibody (NP‐αCD137 Ab‐F4/80) effectively suppresses BRCA BoMet (Table 1) [134].

### 3.3 Metabolic Molecules and Products

#### 3.3.1 Arachidonic Acid

AA significantly upregulated the expression of inducible nitric oxide synthase in human osteoblast‐like cells, consequently enhancing osteoclast activity [137]. Osteoclast‐secreted lipids specifically promote the proliferation and migration of BRCA cells with a propensity for BoMet but not in normal mammary epithelial cells [138]. The content of PUFAs, including AA and eicosapentaenoic acid, secreted by mature osteoclasts is six‐fold higher than that secreted by undifferentiated precursors [138]. AA directly facilitates the migration of BRCA cells and inhibits their apoptosis.

#### 3.3.2 Acyl‐Coenzyme A Binding Protein

Teng et al. conducted an in vivo screening using a CRISPRa‐guided RNA sublibrary and identified the intracellular protein acyl‐coenzyme A binding protein (ACBP) as a BoMet‐promoting gene. Mechanistically, ACBP enhances FA oxidation (FAO) and BoMet colonization through its acyl‐CoA‐binding activity. In the presence of long‐chain FAs, ACBP‐mediated FAO supplies ATP and NADPH, which protect against lipid peroxidation and ferroptosis [139].

#### 3.3.3 FA‐Binding Proteins

The FABP family comprises at least nine homologous proteins with similar structures and tissue‐specific distributions. FABPs primarily (1) facilitate the solubilization, transport, and metabolism of FAs; (2) interact with membranes and intracellular proteins; and (3) regulate tissue‐ and cell‐specific lipid responses [140]. After crossing the membrane, FAs bind to cytosolic FABPs before entering metabolic or signaling pathways. BoMet studies have focused mainly on FABP4, which is predominantly expressed in adipocytes, macrophages, endothelial cells, and cancer cells. Its transcription is regulated by FAs, PPARγ agonists, and insulin [141]. Accumulating evidence has demonstrated that FABP4 promotes tumor cell invasion and metastasis in BRCA and PCa by enhancing FA uptake, activating EMT, and inducting of prometastatic cytokine secretion [142]. Furthermore, elevated FABP4 expression in TAMs enhances lipid storage and lipolysis, thereby generating energy‐rich FAs that fuel cancer cell proliferation, migration, and invasion. Additionally, FABP4^+^ macrophages promote obesity‐associated tumor progression through the concurrent activation of the NLRP3 inflammasome and the secretion of the key proinflammatory cytokine IL‐1β [142]. Herroon et al. reported that FABP4 is significantly overexpressed in prostate skeletal tumors [62]. Lipids supplied by BMAs induce the upregulation of FABP4, IL‐1β, and HMOX‐1 in metastatic cancer cells. Additionally, the interaction between FABP4 and PPARγ further enhances the invasiveness of cancer cells within the BME. The FABP4 inhibitor BMS309403 significantly suppresses PCa cell invasion induced by adipocyte‐conditioned medium [62].

#### 3.3.4 Lysophosphatidic Acid

Glycerol‐3‐phosphate acyltransferase catalyzes the binding of long‐chain FAs to glycerol‐3‐phosphate to produce lysophosphatidic acid (LPA). MDA‐B02 BRCA cells are a subclone of human MDA‐MB‐231 cells extracted from a BALB/c nude mouse model that exhibits high bone tropism and cause osteolytic lesions [143]. Overexpression of the LPA receptor LPA1 in MDA‐B02 cells enhances proliferation both in vitro and in vivo and increases the secretion of osteoclast‐promoting cytokines [144]. LPA directly interacts with osteoclasts and functions similarly to M‐CSF and RANKL in promoting osteoclast differentiation [145]. LPA also promotes osteoblast proliferation [146]. As a paracrine factor present in BME during osteolytic metastasis, LPA prevents osteoblast apoptosis through PI3K‐dependent signaling [147]. In addition, LPA induces cytoskeletal rearrangement and cell migration by activating the Rho/ROCK pathway [148]. LPA synergizes with VitD3 to enhance osteoblast‐like MG63 cell differentiation and bone marrow progenitor osteogenesis [145]. Metastatic BRCA cells induce platelet aggregation with activated platelets and release bioactive LPA. Reducing platelet count pharmacologically lowers circulating LPA levels and suppresses osteolytic BoMet [144]. Furthermore, LPA promotes platelet aggregation and activation, establishing a positive feedback loop between activated platelets and LPA at BoMet sites [149].

#### 3.3.5 Phospholipase D2

Phospholipase D2 (PLD2) is one of the key isoforms of the phospholipase D family, and its primary function is to hydrolyze phosphatidylcholine (PC) to produce phosphatidic acid (PA) and choline. Tumor‐derived exosomes communicate with cells in distant premetastatic organ sites by forming a tumor‐favorable microenvironment [150]. PLD2 in PCa cells stimulates exosome secretion and promotes osteoblast activity, thereby promoting PCa BoMet [151].

#### 3.3.6 Lysophosphatidylcholine

In the de novo synthesis pathway, lysophosphatidic acid acyltransferase transfers the acyl chain of acyl‐CoA to LPA, forming PA. PA is then converted to diacylglycerol, the direct precursor of PC. Increased PC synthesis and elevated levels of choline‐cycle metabolites are significant markers of cancer progression [152]. After synthesis, PC undergoes remodeling, producing lysophosphatidylcholine (LPC) via the cleavage of an acyl chain [153]. The levels of LPC 18:0 and LPC 16:0 are significantly decreased in osteoclastic lipids [138]. Both LPC 18:0 and LPC 16:0 suppress BRCA proliferation and migration while promoting apoptosis, with LPC 18:0 having stronger effects. LPC can be reconverted to PC by LPC acyltransferase or converted to LPA by autotaxin. In conclusion, glycerophospholipids play a pivotal role in BoMet and warrant further investigation.

#### 3.3.7 Sphingosine 1‐Phosphate

Basal catabolism of sphingolipids occurs in the lysosome, where ceramide is deacylated to produce sphingosine, which is phosphorylated into sphingosine 1‐phosphate (S1P) by sphingosine kinase 1 (SPHK1) and sphingosine kinase 2 (SPHK2) [154].

RANKL induces the upregulation of SPHK1 in bone marrow‐derived macrophage monocultures, enhancing S1P biosynthesis and secretion. Conversely, SPHK1 overexpression in bone marrow‐derived macrophage suppresses osteoclastogenesis through modulation of p38 and ERK signaling, accompanied by reduced expression of nuclear factor of activated T‐cells cytoplasmic 1 (NFATc1) and c‐Fos. These findings demonstrate that RANKL‐induced S1P establishes a negative feedback mechanism in bone marrow‐derived macrophage monocultures [155]. In contrast, exogenous S1P administration in bone marrow‐derived macrophage/osteoblast cocultures upregulates RANKL expression and promotes osteoclastogenesis via the cyclooxygenase (COX)‐2/PGE2 pathway. Furthermore, S1P promotes osteoblast migration and viability. In T‐cells experiments, S1P induces chemotaxis and elevates RANKL production. Thus, secreted S1P recruits and activates both osteoblasts and T cells, thereby augmenting osteoclast differentiation [155]. S1P signaling through its receptors sphingosine‐1‐phosphate receptor 1 (S1PR1) and sphingosine‐1‐phosphate receptor 2 (S1PR2) regulates osteoclast precursor mobilization. Loss of S1PR1 in osteoclasts/monocytes reduces bone density, whereas S1PR2 deficiency increases bone density and decreases osteoclast activity [156, 157]. The S1PR2 antagonist JTE013 limits osteoclast activity and reverses bone density loss in a RANKL‐induced osteoporosis mouse model [158]. S1PR1 promotes chemotaxis of osteoclast precursors back into the circulation, whereas S1PR2 drives chemorepulsion, attracting osteoclast precursors to bone where S1P concentrations are lower [159]. In osteoblasts, S1PR1 expression increases during BMP 2‐driven osteoblast differentiation, whereas S1PR2 expression decreases [160]. S1P enhances precursor osteoblasts migration through the S1PR1/JAK1/STAT3 and S1PR2/FAK/PI3K/AKT pathways [161]. S1P primarily exerts its mitogenic effect through functional Gi proteins and p42/44 MAP kinases [162]. Moreover, S1P/S1PRs also regulate immune cell migration, underscoring the critical role of S1P in bone homeostasis [163].

## 4 BoMet From Different Origins and Regulation

Although bone is a common site of metastasis for many malignancies, certain types of cancer have a greater propensity to metastasize to bone, especially breast, prostate, lung, and liver cancers. Moreover, BoMet developing from different cancer types often have distinct pathological and physiological characteristics. Identifying the specific mechanisms of BoMet in these cancers not only highlights the differences among them but also provides a theoretical basis for developing targeted drugs against BoMet in various cancers.

### 4.1 Prostate Cancer

PCa typically follows an occult and indolent course, and more than 90% of patients with advanced PCa develop BoMet [103]. Metastatic PCa primarily results in osteoblastic lesions, although osteolytic components are also present. The preferential localization of PCa cells to osteoblast‐rich niches, and their interaction with osteoblasts promotes the transformation of normal osteocytes into cancer‐associated osteoblasts (CAOs), thereby establishing a microenvironment that supports metastatic colonization. Despite increased osteoblast activity, the organization of the bone matrix is disrupted, weakening the bone microstructure, reducing mechanical strength and stiffness, and ultimately increases fracture susceptibility [103]. Elevated serum levels of osteoblast markers serve as predictors of skeletal complications and are associated with poor OS in patients with PCa [164].

**FIGURE 3:**
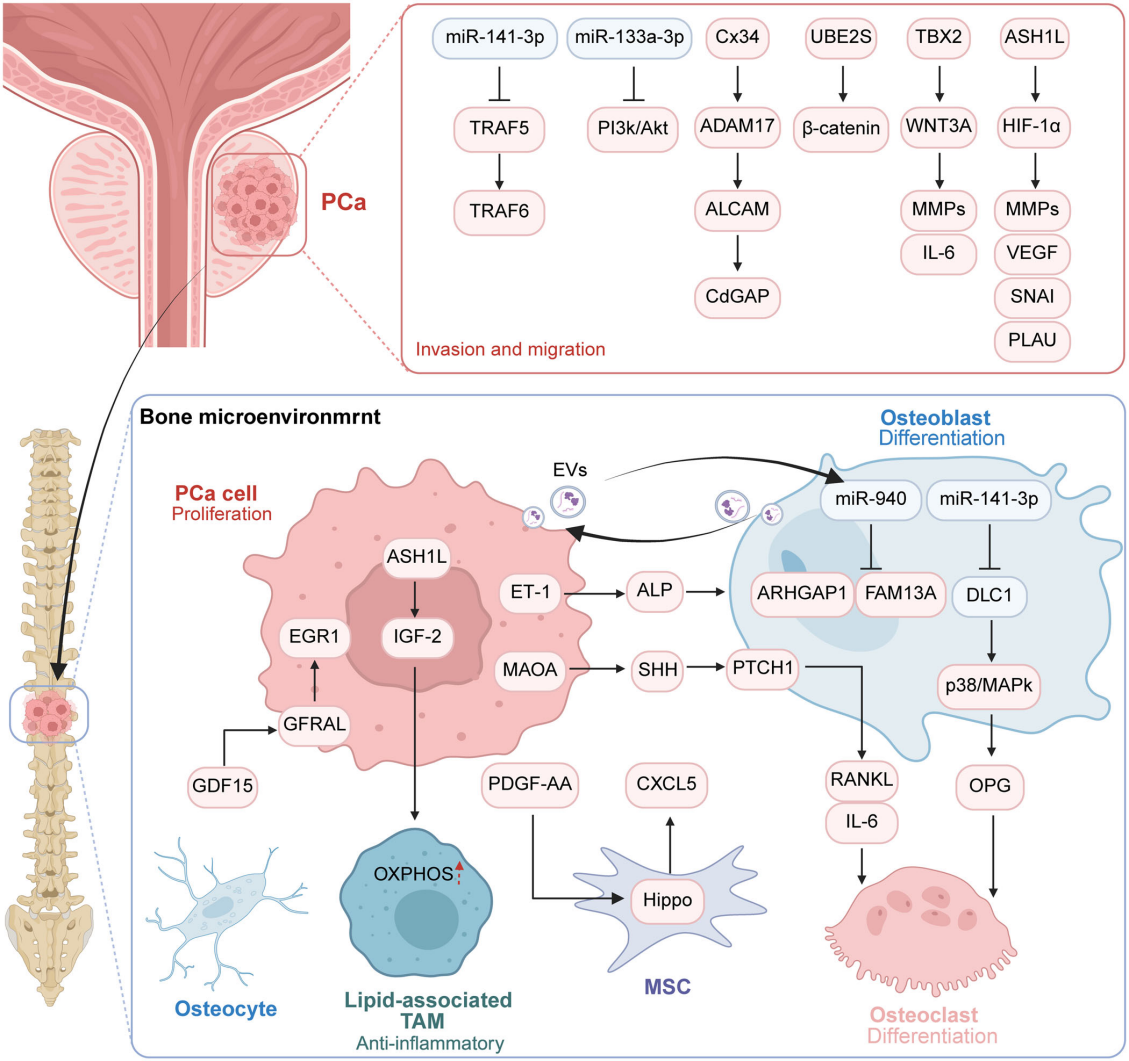
Cellular and molecular interactions in the primary prostate cancer and bone metastatic microenvironment. During the initiation and progression of primary prostate cancer, malignant cells enhance their invasive and metastatic potential through multiple mechanisms. These include activation of the PI3K/AKT pathway and cytokine‐mediated ECM remodeling by MMPs and IL‐6, which collectively facilitate tumor cell infiltration. Within the bone microenvironment, PCa cells secrete factors such as ALP to promote osteoblast differentiation. Conversely, they can modulate osteoclast activity via osteoblast‐mediated signaling, leading to bone matrix resorption that supports tumor growth at metastatic sites. Furthermore, secreted proteins from osteoblasts, osteocytes, and mesenchymal stem cells can directly stimulate PCa cell proliferation, thereby facilitating tumor expansion within the bone. ADAM17, A disintegrin and metalloproteinase 17; ALCAM, activated leukocyte cell adhesion molecule; ALP, alkaline phosphatase; ARHGAP1, Rho GTPase activating protein 1; ASH1L, ASH1 like histone lysine methyltransferase; EGR1, early growth response 1; EVs, extracellular vesicles; GDF15, growth differentiation factor 15; GFRAL, GDNF family receptor alpha‐like; HIF‐1α, hypoxia‐inducible factor‐1α; MSC, mesenchymal stem cell; OXPHOS, oxidative phosphorylation; PCa, prostate cancer; PTCH1, patched 1; SHH, Sonic Hedgehog; TAM, tumor‐associated macrophage; TBX2, T‐box transcription factor 2; TRAF, tumor necrosis factor receptor‐associated factor; UBE2S, ubiquitin conjugating enzyme E2 S. Created with BioRender.com.

As previously stated, the metastatic cascade requires cancer cells to first detach from the primary tumor, undergo local intravascular migration into the blood vessels, and ultimately disseminate to distant organs, including bone. Consequently, the molecules governing tumor cell detachment from the primary tumor merit a detailed discussion. For instance, activated leukocyte cell adhesion molecule (ALCAM) mediates both homotypic and heterotypic cell adhesion in a calcium‐independent manner [165]. ALCAM undergoes proteolytic cleavage at the cell surface by disintegrin and metalloproteinase 17 (ADAM17), resulting in ectodomain shedding. Knockdown of ALCAM in PCa cells reduces bone dissemination and tumor growth in bones [165]. Besides, elevated expression of CdGAP is associated with an increased risk of BoMet in patients with PCa, likely because of its role in regulating EMT in PCa cells [166]. T‐box transcription factor 2 expression was elevated in PCa and BoMet lesions of xenograft mouse models. This transcription factor drives WNT3A expression, consequently upregulating downstream effectors, including MMP2, MMP9, and IL‐6, which collectively enhance migration, invasion, and BoMet in PCa [102]. Moreover, ASH1‐like histone lysine methyltransferase is amplified and overexpressed in metastatic PCa cells, where it catalyzes H3K4me3 and H3K36me2/3 deposition on hypoxia‐inducible factor‐1α (HIF‐1α) target prometastatic genes (e.g., MMPs, VEGF, SNAI, PLAU), thereby enhancing cellular invasiveness [167]. The ubiquitin‐conjugating enzyme E2 S (UBE2S) mediates p16 degradation specifically via K11‐linked ubiquitination, promoting G1/S phase transition in PCa cells. Furthermore, UBE2S stabilizes β‐catenin through K11‐linked ubiquitination, contributing to enhanced migration and invasion of PCa cells during BoMet. Targeting UBE2S with cephalomannine suppressed PCa cell proliferation and invasion in vitro and inhibited PCa BoMet in vivo. Furthermore, the ASH1‐like histone lysine methyltransferase–IGF‐2 axis in metastatic PCa cells induces the formation of lipid‐associated TAMs and sustains their protumorigenic and anti‐inflammatory phenotype via enhanced oxidative phosphorylation (OXPHOS) [167]. The expression of miR‐133a‐3p and miR‐141‐3p was reduced in PCa tissues, particularly in BoMet tissues. Low miR‐133a‐3p expression was significantly associated with shorter BoMet‐free survival in PCa patients. miR‐133a‐3p suppresses PI3K/AKT signaling by directly targeting EGFR, FGFR1, IGF1R, and MET. Administration of agomir‐133a‐3p markedly inhibited PCa BoMet [114]. The miR‐141‐3p inhibits NF‐κB signaling by directly targeting TNF receptor‐associated factors 5 (TRAF5) and 6 (TRAF6), thereby inhibiting the invasion, migration, and BoMet of PCa cells (Figure 3) [168].

The dynamic crosstalk between PCa cells and the BME has been extensively investigated. Among all the metastatic sites, BoMet lesions exhibit uniquely elevated levels of the gap junction subunit connexin 43 (Cx43), a feature associated with poor survival. Elevated Cx43 expression impairs the cytoskeletal dynamics of CAOs and enhances the migration of PCa cells. Osteoblast‐derived Cx43 mediates calcium transfer to PCa cells, supporting their proliferation and survival within the BoMet niche [103]. PCa cells can mimic the function of bone‐resident cells by releasing molecules (such as osteocalcin, OPN, and BMPs) typically associated with bone formation and metabolism. This disrupts bone homeostasis, facilitates surveillance evasion, and ultimately promotes survival within the BME [103]. ET‐1, which is overexpressed in PCa cells, stimulates osteogenesis and induces alkaline phosphatase (ALP) expression, as well as osteoblastic BoMet [169]. A chemokine screening study revealed that PCa cells stimulate osteocytes to secrete growth differentiation factor 15 (GDF15). Osteocyte‐derived GDF15 engages the GDNF family receptor alpha‐like (GFRAL) on PCa cells to promote the expression of early growth response 1, thereby enhancing BoMet [170]. Expression of monoamine oxidase A in PCa cells initiates the premetastatic niche by activating the paracrine Sonic Hedgehog (SHH)–IL‐6–RANKL signaling pathway in osteoblasts [65]. Furthermore, PCa cells communicate with the BME through the secretion and uptake of extracellular vehicles (EVs). Upon the uptake of PCa cell‐derived EVs delivering miR‐141‐3p, osteoblasts exhibited reduced DLC1 protein expression. This activates the p38 MAPK pathway and subsequently stimulates OPG expression, ultimately suppressing osteoclast activity. EVs carrying miR‐940 have been shown to promote the osteoblastic differentiation of human MSCs by targeting Rho GTPase‐activating protein 1 and FAM13A, thereby facilitating osteoblastic BoMet [171]. Notably, cells within the BME also secrete EVs, which in turn modulate tumor cell activity. For instance, the uptake of osteoblast‐derived EVs by PCa cells has been shown to double their proliferation rate compared with control cultures in EV‐free medium [103]. In bone marrow MSCs, MDA‐9 activates the Hippo signaling pathway to induce CXCL5 secretion and promote PCa BoMet [133]. Recent studies have reported a noteworthy phenomenon in which cell fusion during PCa BoMet gives rise to myeloid‐like tumor cells. These hybrid cells were associated with increased rates of BoMet, more extensive skeletal damage, and reduced survival in mouse models [172]. These prometastatic effects are partly attributed to the enhanced EMT phenotype in myeloid‐like tumor cells as well as their capacity to recruit myeloid cells into the BME and polarize them into tumor‐promoting N2 neutrophils or M2 macrophages, thereby establishing an immunosuppressive microenvironment. Moreover, these myeloid‐like tumor hybrid cells exhibit resistance to docetaxel (DTX) and ferroptosis‐inducing agents while retaining sensitivity to radiotherapy [172].

### 4.2 Breast Cancer

BRCA has surpassed lung cancer as the most common malignancy worldwide and is the fifth leading cause of cancer‐related mortality [173]. The skeleton is the most frequent site of BRCA metastasis; approximately 37.5% of patients with Stage IV disease present with BoMet at initial diagnosis, and skeletal involvement is observed in nearly 70% of women who succumb to BRCA [120]. In addition, lymph node involvement and a larger tumor size at diagnosis are established risk factors associated with an increased propensity for skeletal dissemination [120].

**FIGURE 4:**
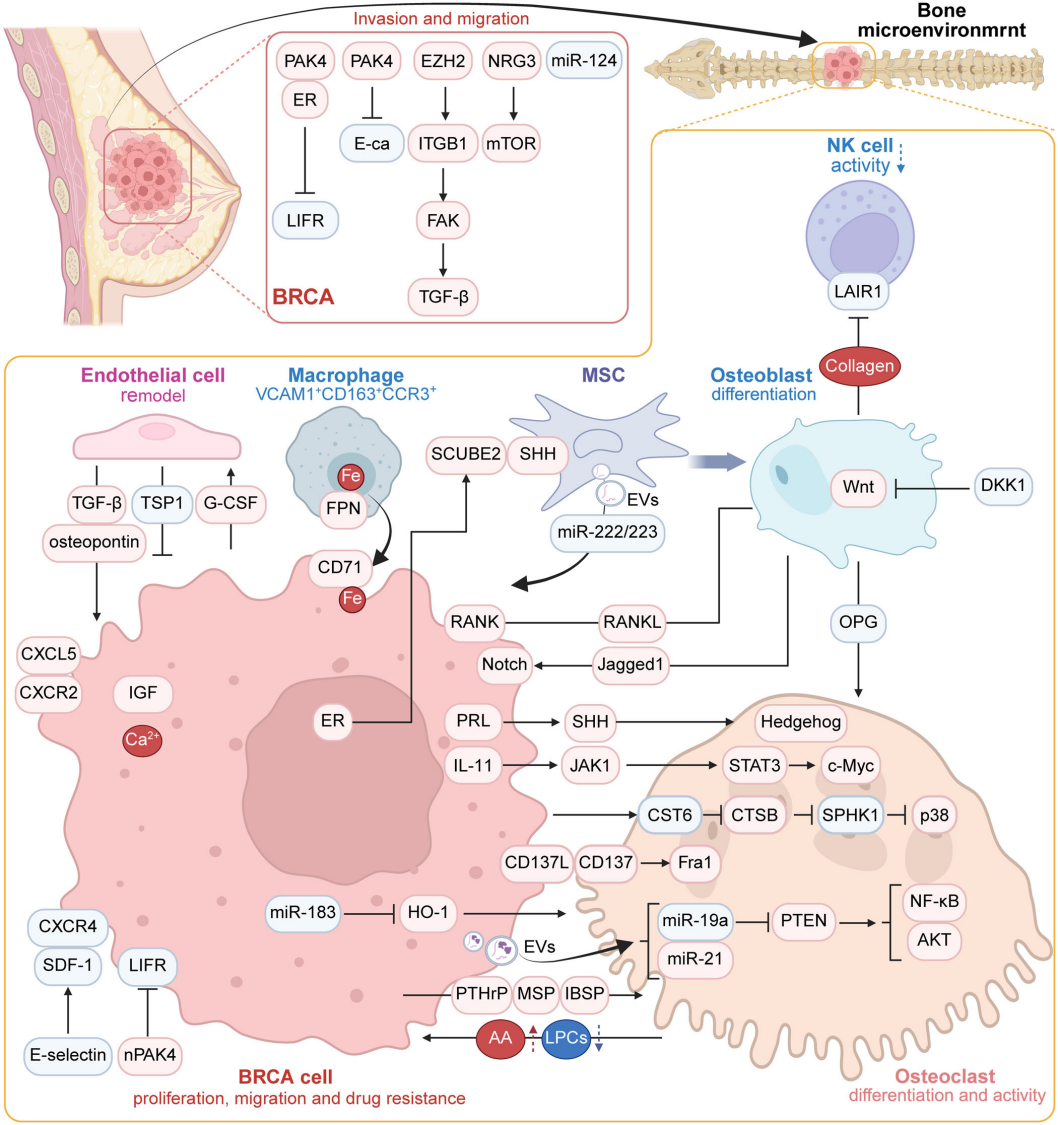
Molecular mechanisms in primary breast tumors and bone metastases niche. Within bone‐metastasizing primary breast tumors, multiple mechanisms facilitate local invasion and dissemination. At the bone metastatic site, breast cancer cells promote osteoclast differentiation through various pathways. Reciprocally, osteoclasts enhance cancer cell proliferation, metastatic potential, and therapy resistance, establishing a vicious cycle. This feed‐forward loop is further orchestrated by other bone stromal components, including endothelial cells, macrophages, mesenchymal stem cells, osteoblasts, and immune cells. AA, arachidonic acid; BRCA, breast cancer; CST6, cystatin E/M; CTSB, cysteine protease cathepsin B; ER, estrogen receptor; FPN, ferroportin; G‐CSF, granulocyte colony‐stimulating factor; IBSP, integrin‐binding sialoprotein; LIFR, leukemia inhibitory factor receptor; LPC, lysophosphatidylcholine; MSP, macrophage‐stimulating protein; NAT1, N‐acetyltransferase 1; NK, natural killer; nPAK4, nuclear p21‐activated kinase 4; NRG3, neuregulin 3; PAK4, p21 activated kinase 4; PRL, prolactin; SCUBE2, signal peptide CUB domain and EGF like domain containing 2; SPHK1, sphingosine kinase 1; TSP‐1, thrombospondin‐1. Created with BioRender.com.

Estrogens bind to the nuclear receptors ER alpha (ERα) and ERβ, which function as transcription factors [174]. Clinical studies indicate that, while ER^+^ luminal BRCA generally has a lower risk of metastasis to most distant organs, it exhibits a distinct and pronounced tropism for bone tissue [175, 176]. ER^+^ BRCA cells show enhanced survival and proliferation in bone under estrogen stimulation, a dependency that can be therapeutically targeted by aromatase inhibitors [176]. Bado et al. reported a positive correlation between the size of the BoMet niche and nuclear ER expression [177]. Compared with primary BRCA, early BoMet exhibited significantly reduced estrogen uptake; however, this difference gradually diminished during the later stages of BoMet. This is attributed to the osteogenic niche enhancing the phenotypic plasticity of metastatic ER^+^ BRCA cells via EZH2‐mediated epigenomic reprogramming [177]. Expression of miR‐19a and integrin‐binding sialoprotein (IBSP) is upregulated in EVs from ER^+^ osteotropic BRCA cells, promoting BoMet [178]. In addition, Wu et al. reported that the ER‐induced secreted protein signal peptide CUB domain and EGF‐like domain containing 2 (SCUBE2) were upregulated in luminal BRCA and promoted BoMet [175]. Single‐cell sequencing revealed that SCUBE2 is associated with the enrichment of osteoblast populations in the BoMet niche. Mechanistically, SCUBE2 facilitates the release of membrane‐anchored SHH from tumor cells, which activates the Hedgehog signaling pathway in MSCs and promotes their differentiation into osteoblasts. These newly formed osteoblasts then deposit collagen and suppress the activity of NK cell through inhibitory LAIR1 signaling, ultimately enhancing tumor colonization within BME [175]. The 17β‐estradiol (E2) stimulation promotes the formation of a p21‐activated kinase 4 (PAK4)–ERα complex that represses the ERα target gene leukemia inhibitory factor receptor (LIFR) to drive metastasis. PAK4 also facilitates cancer cell invasion by suppressing E‐cadherin and inducing EMT [179]. In BRCA cells, the transcription factor EZH2 upregulates integrin β1 (ITGB1) expression, leading to subsequent activation of its downstream effector FAK. Activated FAK subsequently phosphorylates TGF‐βRI and stabilizes its association with TGF‐βRII, consequently leading to the activation of the TGF‐β signaling pathway (Figure 4) [108]. The estrogen‐related receptors (ERRs), comprising ERRα, ERRβ, and ERRγ, constitute a subfamily of nuclear receptors that share structural homology with ERs in their DNA‐binding domains but exhibit only moderate similarity in their ligand‐binding pockets [180, 181]. Accumulating studies have revealed contradictory roles of ERRα in regulating BoMet. ERRα promotes osteoblast differentiation and proliferation by upregulating bone sialoprotein, ALP, OPN and glutaminase‐mediated glutaminolysis while inhibiting PPAR and adaptor protein 2. Additionally, ERRα interacts with PPAR‐γ coactivator‐1 to promote osteoclast formation by synergistically regulating the expression of OPN and β3‐integrin, as well as mitochondrial metabolism and oxidative processes [180]. Furthermore, ERRα promotes BoMet by upregulating RANKL, which activates mTOR signaling in BRCA cells. The ERRs inhibitor C29 suppresses both primary tumorigenesis and BoMet [182]. However, ERRα also upregulates OPG, thereby inhibiting osteoclast differentiation and restricting tumorigenesis in bone [183]. In addition, ERRα induces the expression of chemokines in the BME and downregulates TGF‐β, inhibiting the growth of BRCA cells following their anchoring in the bone [184]. Thus, the precise function of ERRα in BoMet requires further investigation to determine its potentially distinct roles during different phases of the metastatic cascade, as well as in osteolytic versus osteoblastic lesions.

The clinical interval between the diagnosis of primary BRCA and the detection of overt BoMet can span several years, indicating the potential for prolonged tumor cell dormancy. Endothelial cells expressing thrombospondin‐1 (TSP‐1) maintain BRCA cell quiescence [120]. Furthermore, EVs from bone marrow MSCs can deliver miR‐222/223 to BRCA cells, reinforcing the dormant state [185]. Reactivation of these dormant cells involves remodeling of the perivascular niche. Sprouting endothelial cells show reduced TSP‐1 expression and secrete OPN and TGF‐β, which stimulate dormant tumor cell proliferation [120]. Dormant BRCA cells were predominantly localized within perivascular niches enriched in E‐selectin and stromal cell‐derived factor 1 (SDF‐1). E‐selectin facilitates the transendothelial migration of BRCA cells into the bone marrow by modulating the sinusoidal endothelium, whereas SDF‐1 anchors tumor cells within the BME by interacting with its receptor, CXCR4. Blocking the SDF‐1/CXCR4 axis mobilizes dormant micrometastatic foci into circulation, thereby removing BRCA cells from the protective bone marrow niche and preventing their reactivation as recurrent disease [46]. Furthermore, nuclear p21‐activated kinase 4 suppresses dormancy in bone‐metastasized BRCA cells by targeting the LIFR–STAT3 signaling axis [186].

The BoMet niche is shaped by BRCA cells through multiple mechanisms that influence its key cellular constituents. The interaction between CD137L on BRCA cells and CD137 on macrophages induces Fra1 expression in macrophages, promoting their migration to the BME and their differentiation into osteoclasts [134]. Cystatin E/M derived from BRCA cells is internalized by osteoclasts via endocytosis, where it inhibits the cysteine protease cathepsin B (CTSB), resulting in the accumulation of its hydrolytic substrate SPHK1. Elevated SPHK1 suppress osteoclast maturation and BoMet by inhibiting the RANKL‐induced activation of the p38 MAPK pathway [187]. Prolactin (PRL) promotes bone resorption by inducing SHH secretion from BRCA cells, which activates the Hedgehog pathway in osteoclasts [188]. Moreover, highly bone‐tropic BRCA cells exhibit elevated IL‐11 expression, which activates the JAK1/STAT3 signaling pathway in bone marrow macrophages to upregulate c‐Myc, thereby promoting RANKL‐independent osteoclastogenesis [124]. Expression of miR‐124 was significantly downregulated in BRCA tissues compared with that in normal breast tissues and was further reduction in BoMet samples. Downregulation of miRNA‐124 correlates with increased invasiveness of BRCA cells and shortened BoMet‐free survival. Mechanistically, miR‐124 downregulation stimulates the production of pro‐osteoclastic cytokines, thereby promoting bone resorption [189]. Furthermore, miR‐183 derived from BRCA cells suppresses heme oxygenase‐1, thereby enhancing osteoclastogenesis [190]. Wu et al. identified 157 miRNAs that were significantly upregulated in EVs secreted by bone‐tropic ER^+^ BRCA cells. Among these, three miRNAs (miR‐19a, miR‐133b, and miR‐576‐5p) were markedly elevated in the plasma of patients with ER^+^ BRCA [178]. Notably, only the expression level of miR‐19a showed a significant correlation with BoMet‐free survival and was selectively elevated in BoMet lesions. Mechanistically, EV‐derived miR‐19a is internalized by macrophages, where it targeted phosphatase and tensin homolog (PTEN) to repress NF‐κB and AKT signaling, thereby suppressing osteoclast differentiation and BoMet. Moreover, the IBSP secreted by BRCA cells recruits osteoclasts and acts synergistically with miR‐19a to promote BoMet [178]. In a parallel pathway, miR‐21 in EVs derived from BRCA cells acts directly on osteoclasts, thereby promoting osteoclast differentiation and potentiating bone resorption [191]. Secretion of PTHrP by BRCA cells in the BME promotes bone resorption by osteoclasts [192]. By modulating canonical Wnt signaling in osteoblasts, DKK1 suppresses OPG secretion, thereby facilitating osteoclast differentiation and BRCA BoMet [105]. BRCA cells secrete macrophage‐stimulating protein (MSP) that activate the recepteur d'origine nantais (RON) signaling pathway, which promotes the activation and bone‐resorbing capacity of osteoclasts without affecting their differentiation [193]. In ER^+^ BRCA, secreted IBSP recruits preosteoblasts to the metastatic site. Within this preosteoblast‐rich niche, cancer‐derived EVs deliver miR‐19a to preosteoblasts, thereby suppressing PTEN expression. This downregulation of PTEN activates both NF‐κB and AKT signaling pathways, which in turn promotes osteoclast differentiation and enhances bone resorption [178]. Besides. utilizing a spontaneous BRCA BoMet mouse model, researchers observed that DTCs preferentially localize within specific H‐type vessel‐enriched vascular niches, rather than arterial or L‐type sinusoidal vessels. Mechanistically, DTCs remodel the vascular endothelium via G‐CSF secretion, thereby establishing a microenvironment conducive to BoMet [127]. Under physiological conditions, erythroblastic islands (EBIs) expressing VCAM1^+^CD163^+^CCR3^+^ macrophages export recycled heme‐derived iron to erythroblasts via ferroportin, thereby supporting hemoglobin synthesis and erythropoiesis. Upon colonizing the bone marrow, tumor cells hijack iron‐laden VCAM1^+^CD163^+^CCR3^+^ macrophages, diverting them from EBIs and their recycled iron to fuel tumor cell growth and heme synthesis [194]. Under hypoxic stress, tumor cells increase GATA1 expression to upregulate globin genes, thereby enhancing their adaptability to the hypoxic bone niche. These mechanisms deplete iron in erythroblasts, suppress erythropoiesis, and ultimately promote anemia [194].

BME actively promotes the malignant progression of cancer cells. Factors (such as TGF‐β, IGF, and Ca^2^^+^) released during cancer‐mediated bone resorption further enhance cancer cell survival and proliferation [195]. Metabolic reprogramming of osteoclasts elevates prometastatic AA while reducing antimetastatic LPCs, thereby enhancing BRCA cell proliferation, migration, survival, and expression of prometastasis genes [138]. E‐cadherin‐expressing BRCA cells form heterotypic adhesions with N‐cadherin in osteoblasts. Adhesion junctions activate the Akt/mTOR signaling pathway in BRCA cells, stimulating their proliferation and facilitating the development of BoMet [196]. Osteoblast‐derived RANKL attracts RANK‐expressing BRCA cells and induces their migration to the bone niche. Moreover, RANKL signaling upregulates the expression of prometastatic genes (e.g., IL‐11, NCF2, PRG‐1, and MMP‐1) in tumor cells [197]. Under physiological conditions, factors secreted by BME cells act to sequester or neutralize CXCL5, preventing its interaction with cancer cell receptors and thereby maintaining these cells in a dormant state. In contrast, BRCA cells release CXCL5 from inhibitory control, leading to excessive CXCL5–CXCR2 binding, which subsequently stimulates tumor cell proliferation and colonization within the bone [130]. Molecular crosstalk between cancer cells and BME is a key driver of acquired drug resistance. For instance, chemotherapy induces Jagged1 expression in osteoblasts. This ligand then activates Notch signaling in cancer cells, promoting drug resistance [107]. Nevertheless, these interactions represent potential therapeutic targets. A notable example is the gap junctions formed between osteoblasts and cancer cells, which facilitate calcium ion transfer. This intercellular communication unexpectedly confers a specific vulnerability to arsenic trioxide therapy in metastatic cells (Figure 4) [198].

### 4.3 Lung Cancer

The 5‐year survival rate for advanced lung cancer remains below 5%, and more than 90% of lung cancer‐related deaths are attributed to metastatic complications [199]. Bone is one of the most common metastatic sites in lung cancer; approximately 40–48% of patients with advanced lung cancer are initially diagnosed with BoMet at initial presentation [200].

**FIGURE 5:**
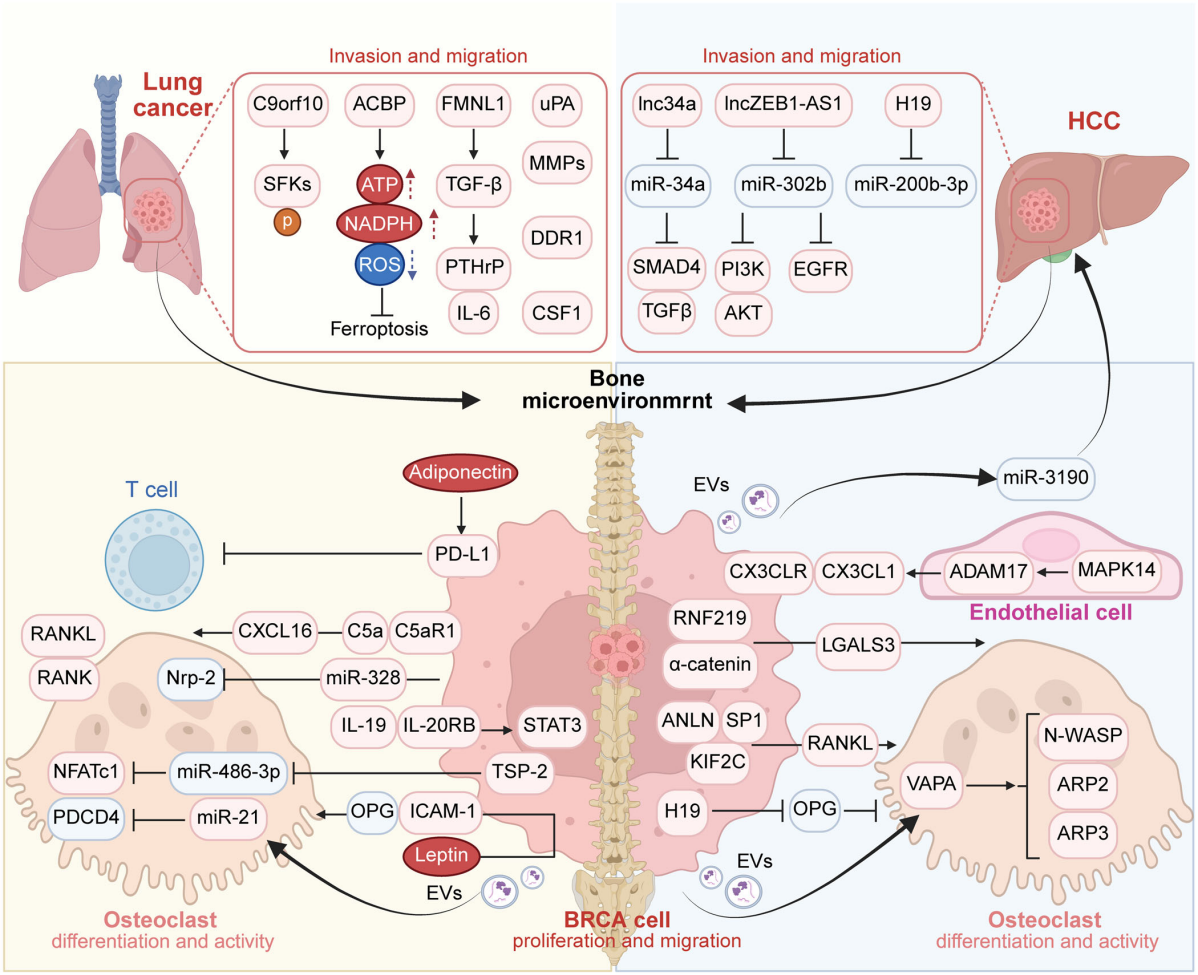
Mechanisms of bone metastasis in lung and liver cancers. Primary lung (left) and liver (right) cancer lesions enhance their invasive and metastatic potential through epithelial–mesenchymal transition and extracellular matrix degradation. Within bone metastases, both lung (left) and liver (right) cancer cells predominantly drive osteoclast differentiation. Lung cancer additionally suppresses antitumor immunity, while HCC‐derived exosomes from bone lesions further stimulate the invasiveness and metastatic capacity of primary hepatic tumors. ACBP, acyl‐coenzyme A binding protein; ADAM17, A disintegrin and metalloproteinase 17; ANLN, anillin; ARP, actin‐related protein; C9orf10, chromosome 9 open reading frame10; EGFR, epidermal growth factor receptor; HCC, hepatocellular carcinoma; ICAM‐1, intercellular adhesion molecule 1; KIF2C, kinesin family member 2C; LGALS3, galectin 3; NFATc1, nuclear factor of activated T‐cells cytoplasmic 1; Nrp‐2, neuropilin 2; PD‐L1, programmed cell death 1 ligand 1; PDCD4, programmed cell death 4; RNF219, ring finger protein 219; ROS, reactive oxygen species; SFKs, Src family kinases; STAT3, signal transducer and activator of transcription 3; TSP‐2, thrombospondin‐2; uPA, plasminogen activator, urokinase; VAPA, VAMP‐associated protein A. Created with BioRender.com.

The highly bone‐tropic lung adenocarcinoma cell line H322L–BO4 was derived via a sequential in vivo selection process involving (1) intracardiac injection of parental H322 cells into nude mice, (2) isolation of bone metastatic lesions, and (3) repeated intracardiac inoculation cycles to derive a bone‐tropic variant [201]. H322L–BO4 cells exhibited significantly elevated levels of chromosome 9 open reading frame 10 (C9orf10), an Src activator, accompanied by increased phosphorylation of downstream Src family kinases (SFKs). Animal studies further demonstrated that the targeted inhibition of C9orf10 effectively suppressed lung adenocarcinoma BoMet [201]. Employing an in vivo CRISPR screen, Teng et al. recently identified ACBP as a key driver of BoMet in human NSCLC cells. Mechanistically, ACBP‐mediated fatty FAO increased the intracellular ATP and NADPH levels. This reductive metabolic state lowers reactive oxygen species, thereby suppressing lipid peroxidation and ferroptosis (Figure 5) [139].

Lung cancer cells secrete MMPs and urokinase plasminogen activators, which are enzymes that specifically target and degrade bone matrix components, thereby facilitating osteolytic destruction and bone invasion [15]. Approximately 70% of BoMet in lung cancer is osteolytic [15]. The inhibition of discoidin domain receptor 1 impairs tumor cell survival and disrupts tumor‐BME crosstalk during the colonization phase, thereby suppressing osteolytic BoMet [202]. Suppression of endogenous CSF1 expression in lung cancer cells significantly inhibits the development of osteolytic BoMet [126]. The expression of formin‐like protein 1 (FMNL1) is significantly upregulated in primary NSCLC tissues and is further elevated in BoMet lesions [203]. FMNL1 promoted NSCLC cells proliferation, migration, and invasion. Furthermore, FMNL1 enhances TGF‐β signaling to drive BoMet [203]. TGF‐β promotes BoMet by inducing the expression of PTHrP and IL‐6, whereas inhibition of Notch3 suppresses this TGF‐β‐mediated effect [111]. Accumulating evidence indicates that lung cancer cells enhance osteoclast differentiation at the BoMet foci through diverse mechanisms. For instance, lung cancer cells overexpressing C5aR1 upregulate the expression and secretion of CXCL16 [118]. TSP‐2 promotes osteoclast formation and facilitates BoMet by suppressing miR‐486‐3p expression in osteoclast precursor macrophages, leading to the transactivation of NFATc1 [204]. Moreover, lung adenocarcinoma cells promote bone resorption by secreting EVs enriched with miR‐328, which suppress the expression of neuronal cell adhesion molecule 2 [205]. Tumor‐derived miR‐21 in EVs downregulates programmed cell death 4 expression in bone marrow monocytes [206]. Collectively, these mechanisms promote osteoclast differentiation. Leptin exhibits dual regulatory effects on bone remodeling: (1) It stimulates soluble intercellular adhesion molecule 1 production that synergizes with RANKL activation to promote osteoclastogenesis, while simultaneously (2) suppressing RANKL expression in osteoblasts to inhibit osteoclast differentiation. Additionally, leptin upregulates OPG expression, thereby preventing RANKL/RANK interactions and indirectly attenuating bone resorption [15]. In addition to its well‐established role in driving osteoclast differentiation, RANKL also exerts direct effects on lung cancer cells. By activating its receptor, RANK, on tumor cells, RANKL promotes cancer cell proliferation and survival, while concurrently suppressing apoptosis [207]. Lung cancer cells stimulate osteoclasts to secrete IL‐19, which in turn binds to IL‐20RB receptors on tumor cells, activating the downstream JAK1–STAT3 signaling pathway and enhancing the proliferative capacity of cancer cells within the BME [125]. Osteoblastic BoMet is relatively rare in patients with lung cancer. However, osteoblastic metastasis in NSCLC is typically associated with adenocarcinoma. Furthermore, a significant proportion of these patients harbored ROS1 rearrangements [208]. Besides, in vitro studies demonstrate that HIF‐1α can suppress osteogenic differentiation by upregulating semaphorin 4D. However, direct evidence linking the HIF‐1α‐mediated regulatory axis to lung cancer BoMet remains elusive [209]. Patients with lung cancer and BoMet exhibit significantly elevated levels of adiponectin and its receptors compared with lung cancer patients without BoMet [210]. Adiponectin upregulates the expression of programmed cell death 1 ligand 1 in tumor cells, thereby impairing immune cell‐mediated tumor cytotoxicity (Figure 5) [211].

### 4.4 Hepatocellular Carcinoma

The clinicopathological features of BoMet in hepatocellular carcinoma (HCC) remain less characterized than those in other malignancies, partly because of its historically low diagnostic frequency. Recent advances in diagnostic imaging and multidisciplinary treatment approaches have improved the clinical management and prognosis of patients with HCC. Consequently, as the OS increases, the reported incidence of BoMet in HCC has steadily increase [23]. Indeed, bone has emerged as the second most common site of HCC metastasis, accounting for approximately 30% of extrahepatic cases, surpassed by lung metastasis [23]. HCC BoMet exhibits a predilection for the axial skeleton, potentially attributable to portal hypertension and the consequent development of collateral networks via the spinal venous system [212]. Histologically, HCC BoMet was predominantly osteolytic (82.44%), with osteoblastic (9.76%) and mixed (7.80%) lesions being less frequent [23].

In addition to the established mechanisms that enhance the intrinsic invasiveness of HCC cells, such as EMT and angiogenesis, recent studies have uncovered multiple novel regulatory pathways governing HCC BoMet (Figure 5). Patients with HCC with elevated ring finger protein 219 (RNF219) expression exhibit shorter progression‐free survival (PFS) than those with lower RNF219 expression. Mechanistically, hyperactivation of the RNF219/α‐catenin/galectin 3 (LGALS3) axis in HCC cells enhances pro‐osteolytic crosstalk with preosteoclasts, thereby driving osteoclastogenesis and BoMet [213]. Noncoding RNAs have emerged as critical contributors to BoMet in HCC. Lnc34a acts as a molecular sponge for miR‐34a, and its action involves the recruitment of DNMT3A/PHB2 and HDAC1 complexes. The downregulation of miR‐34a enhances cancer cell migration and invasion and promotes the transcription of osteotropic genes via SMAD4/TGF‐β signaling [214]. The long noncoding RNA ZEB1–AS1 functions as a molecular sponge for miR‐302b, leading to the activation of the PI3K/AKT pathway and upregulation of EGFR, thereby promoting BoMet in HCC [215]. LncRNA H19 promotes the metastatic dissemination of HCC cells by inducing EMT by sponging miR‐200b‐3p. Concurrently, H19 suppresses p38 MAPK signaling to reduce OPG expression, thereby exacerbating osteolysis at the BoMet site [216]. VAMP‐associated protein A‐enriched large oncosomes derived from HCC showed significantly enhanced bone tropism. This effect is mediated by the N‐WASP/Arp2/Arp3 signaling pathway, which drives osteoclast fusion and activation, thereby fostering a prometastatic niche conducive to bone colonization [217]. Recent research has identified that EVs derived from bone metastatic HCC home to primary HCC tumors and accelerate their progression. Mechanistically, miR‐3190 expression was elevated in bone‐tropic HCC cells, resulting in selective packaging into EVs. Upon internalization by primary HCC cells, the delivered miR‐3190 suppresses the expression of the RNA demethylase alkB homolog 5, which in turn activates a prometastatic gene program, thereby instigating a feed‐forward metastatic cascade [218]. Hepatic cancer‐associated fibroblasts secrete a repertoire of chemokines, including CCL2, CCL5, CCL7, and CXCL16, which activate both Hedgehog and TGF‐β signaling pathways in tumor cells, thereby driving the metastatic spread of HCC to bone [219]. Hyperactivation of p38 in bone marrow endothelial cells promotes spinal metastasis of CX3CR1‐high HCC cells by inducing the ADAM17–CX3CL1 axis, which concomitantly activates PI3K/AKT and RHOA/ROCK signaling [220]. Anillin (ANLN) mRNA stability is enhanced in HCC cells via METTL3‐ and YTHDF1‐mediated m^6^A modifications. Nuclear ANLN forms a complex with the transcription factor SP1 to upregulate the expression of kinesin family member 2C, which in turn activates the mTORC1 pathway. This ANLN/SP1/kinesin family member 2C/mTORC1 signaling cascade perturbs the RANKL/OPG balance within the BME, ultimately facilitating osteolytic invasion (Figure 5) [221].

## 5 Diagnosis

When patients with cancer present with symptoms, such as bone pain, pathological fractures, elevated ALP, or hypercalcemia, timely imaging evaluation is essential to identify potential BoMet. Several imaging modalities are currently used, each with distinct advantages and inherent limitations. Conventional X‐radiation (X‐rays) remain the most fundamental and widely used method, providing the ability to localize lesions and assess their relationships with adjacent bone structures and joints. While inexpensive and accessible, their sensitivity is relatively low, particularly for detecting early stage or small lesions [222]. Bone scintigraphy (BS) or bone scanning relies on the preferential uptake of radiolabeled phosphonates in skeletal regions. This technique exhibits high sensitivity for detecting osteoblastic or mixed metastases, similar to those commonly associated with PCa [23]. However, its sensitivity is limited to malignancies predominantly characterized by osteolytic destruction, including renal cell carcinoma, lymphoma, and HCC. Additionally, BS cannot reliably differentiate metastatic progression from treatment‐related “flare phenomenon” [23]. Computed tomography (CT) provides higher sensitivity and specificity than X‐rays, especially in regions with complex anatomy. CT enables visualization of the size, location, and extent of BoMet, as well as posttreatment changes, such as bone destruction, repair, and calcification. Consequently, CT is often regarded as the preferred modality for evaluating therapeutic responses. However, CT has limited sensitivity for distinguishing focal osteoporosis from small, disseminated lesions [223]. Whole‐body bone scans using emission CT (ECT) provide a comprehensive assessment of the skeleton and are recommended as the first‐line imaging technique for asymptomatic patients. Nevertheless, ECT cannot differentiate osteolytic lesions from osteoblastic lesions and fails to clearly depict the degree of bone destruction [224]. Magnetic resonance imaging (MRI) offers superior soft tissue contrast and provides more accurate information regarding the location, extent, and soft tissue involvement of bone lesions. MRI also avoids radiation exposure; however, because the cortical bone appears as a dark signal on both T1‐ and T2‐weighted sequences, it cannot reliably reflect bone repair or cortical damage [120].

### 5.1 Positron Emission Tomography/CT

Positron emission tomography (PET)/CT visualizes most malignant tumors using positron‐emitting radiopharmaceuticals and uniquely integrates biological information from PET with anatomical data from CT in a single examination. A diverse array of PET radiotracers targeting distinct biological processes have been utilized for diagnosing PCa; these include increased glucose metabolism [^18^F‐fluorodeoxyglucose (^18^F‐FDG)], amino acid transport and protein synthesis (^11^C‐methionine), FA synthesis (^11^C‐acetate), phospholipid cell membrane synthesis (^11^C‐ and ^18^F‐choline), AR (16β‐^18^F‐fluoro‐5α‐dihydrotestosterone), prostate‐specific membrane antigen targeting (^68^Ga), and osteoblastic activity in bone metastatic lesions (^18^F‐NaF) [225].

^18^F‐FDG, the most commonly used PET tracer, demonstrates limited diagnostic performance in evaluating patients with PCa. Recently, ^18^F‐FDG PET/CT has been incorporated into diagnostic workups for small‐cell lung cancer, demonstrating superior sensitivity for BoMet detection. Furthermore, a prospective cohort study of patients with small‐cell lung cancer with BoMet demonstrated that ^18^F‐FDG PET/CT exhibited significantly higher sensitivity than conventional ^99^mTc‐methylene diphosphonate (^99^mTc‐MDP) BS [226].

Similar to other ^18^F‐labeled radiotracers, ^18^F‐NaF is synthesized using a cyclotron and has a physical half‐life of approximately 110 min. Its uptake is primarily governed by regional blood flow and osteoblastic activity. Approximately 50% of the administered dose is incorporated into the bone matrix, 30% is distributed within the red blood cells, and the remainder is excreted via urine within 6 h postinjection [225]. ^18^F‐NaF PET/CT reflects increased bone remodeling and is considered a highly sensitive imaging modality for the detection of BoMet in PCa. However, because the tracer can nonspecifically accumulate in degenerative and inflammatory bone conditions, its diagnostic specificity remains limited. Similar to the limitations of other PET modalities, small metastatic lesions may go undetected due to insufficient tracer uptake, especially in the spine. Positive findings on ^18^F‐NaF PET without corresponding sclerotic changes on CT may result from reactive osteoblastic activity associated with osteolytic damage and malignant bone marrow infiltration, as indicated by increased peripheral ^18^F‐NaF uptake around the lesion [225].

Choline uptake is a hallmark of malignant tumor proliferation and has led to the development of ^18^F‐fluorocholine or ^11^C‐fluorocholine by radiolabeling choline with positron‐emitting radionuclides. ^18^F‐fluorocholine is a valuable tool for the early detection of BoMet as it facilitates the exclusion of distant metastases and the evaluation of responses to hormone therapy. Furthermore, as degenerative changes exhibit no affinity for choline, ^18^F‐fluorocholine can effectively differentiate between degenerative and malignant bone lesions. Researchers have suggested that ^18^F‐fluorocholine PET/CT is a valuable tool for preoperative staging to exclude distant metastases, particularly in patients who have not undergone antiandrogen therapy. The sensitivity, specificity, and accuracy of early BoMet detection were 79, 97, and 84%, respectively. According to Beauregard et al., the patient‐based sensitivity of ^18^F‐fluorocholine PET/CT for detecting BoMet was 100%, compared with only 67% for both ^18^F‐FDG PET/CT and conventional imaging [225]. An important interpretive challenge arises in recurrent PCa, particularly in patients treated with hormone therapy, where mild‐to‐moderate heterogeneous reactive bone marrow uptake of ^18^F‐fluorocholine can occur, potentially complicating image analysis. To overcome this limitation, a dual‐timepoint imaging protocol has been proposed. Increased tracer uptake and higher standardized uptake values on delayed images (90–120 min postinjection) are useful diagnostic indicators for distinguishing between malignant and benign lesions [225].

### 5.2 Serum Markers

Concurrently, significant efforts are underway to develop and validate serum‐based predictive models for assessing the risk of BoMet onset and for prognostic stratification. In a large randomized AZURE trial, Brown et al. reported that elevated serum levels of the bone formation marker procollagen Type I N‐terminal propeptide (P1NP) and the resorption marker Type I collagen C‐telopeptide (CTX) or crosslinked C‐telopeptide were associated with an increased risk of BoMet, with P1NP emerging as the most sensitive predictor [227]. Subsequent clinical investigations have corroborated the prognostic value of these markers. An investigation involving 164 patients with untreated Stage I–III BRCA found that a baseline serum P1NP level ≥75 ng/mL predicted a higher risk of BoMet and was associated with shorter OS [228]. A clinical study of lung cancer patients with BoMet revealed significantly elevated serum levels of BME cytokines (including CaN, OPG, PTHrP, and IL‐6) and bone biochemical markers (tP1NP, β‐CTx) compared with healthy controls [229]. The prediction model incorporating OPG, PTHrP, tP1NP, and β‐CTx demonstrated 87.32% diagnostic accuracy (85.7% specificity, 87.5% sensitivity), enabling the detection of BoMet a median of 9.46 months earlier than conventional BS [229]. In a zoledronic acid‐treated mouse model of lung cancer BoMet, PINP showed strong correlations with osteolytic lesions and tumor burden at both the early and late stages of bone colonization, whereas osteocalcin and CTX showed strong correlations only at advanced stages [230]. Researchers screened a panel of 380 human miRNAs via qPCR and identified miR‐193 as the only miRNA whose expression was correlated with the biomarkers P1NP, osteocalcin, and CTX in zoledronic acid‐treated animals. Moreover, miR‐326 expression strongly correlated with both PINP and tumor burden, suggesting its potential as a novel biomarker for monitoring BoMet progression [230]. A retrospective analysis revealed that patients with elevated serum RANKL levels had an 87.5% increased risk of developing BoMet compared with those with lower levels. Consistently, the serum RANKL/OPG ratio is significantly elevated in patients who subsequently develop BoMet [231].

Currently, aberrations in any single biomarker are insufficient to reliably indicate disease progression. For occult BoMet, or when metastatic lesions remain in a dormant state, existing serum biomarkers show limited sensitivity and fail to detect metastasis before it becomes clinically evident. Therefore, there is an urgent need to develop novel biomarkers for BoMet to enable earlier prediction and prevention in clinical practice.

### 5.3 Liquid Biopsy of Blood

Although fine‐needle biopsy remains the standard procedure for diagnosing BoMet and assessing prognosis, it is an invasive procedure that carries inherent risks of complications. Consequently, minimally invasive liquid biopsy of blood is an attractive alternative. Liquid biopsy can provide complementary clinical information by analyzing circulating tumor DNA and exosomes, which are also detectable in the urine. CTCs can be isolated from peripheral blood using immunomagnetic beads based on their CD45^−^, cytokeratin^+^, and EpCAM^+^ phenotype. Patients with persistently detectable CTCs before and after chemotherapy exhibited a higher incidence of BoMet than those who remain consistently CTC‐negative [232]. The molecular profile of CTCs may provide insights into organ tropism and predict the risk of BoMet. Specifically, elevated expression of TFF1, TFF3, and AR mRNA in CTCs correlates with and may predict the development of BoMet [233, 234]. Osteocalcin is a marker of late osteoblast differentiation. Circulating osteocalcin‐positive cells (cOCs) are small mononuclear cells within the peripheral blood mononuclear cell population that express osteocalcin. In animal models, cOCs levels rise significantly during early BoMet but decline with subsequent tumor progression. Clinically, patients with high cOCs levels have significantly shorter BoMet‐free survival than those with low cOC levels. Notably, this cOC elevation is metastasis‐specific as it is not observed in benign bone conditions such as fractures, underscoring its potential for predicting early BoMet [235].

### 5.4 Artificial Intelligence

Artificial intelligence (AI) has considerable potential for medical applications. Zhang et al. developed and validated a fully automated bone lesion detection system (BLDS) using a large retrospective cohort from a specialized cancer center [236]. The performance of the system was subsequently evaluated by radiologists in a randomized crossover study involving cohorts from five major tertiary hospitals. Finally, the BLDS was deployed in a routine clinical workflow across multiple real‐world settings encompassing 54,610 patients. The results demonstrated that the BLDS significantly enhanced the detection of BoMet in CT scans, achieving high sensitivity and accuracy. Furthermore, the BLDS automatically identifies bone lesions throughout the scan field and classifies them into specific types, substantially improving diagnostic performance [236]. Li et al. proposed a multimodal Swin transformer‐based deep learning model that integrates CT imaging and pathological data to predict BoMet risk in patients with lung cancer. This integrated model exhibited high accuracy, sensitivity, and specificity for predicting BoMet risk [210].

## 6 Clinical Management

Contemporary clinical management of BoMet involves a comprehensive strategy targeting both the primary tumor and established skeletal lesions. BoMet remains incurable, with current treatments focusing on palliative care to alleviate cancer‐induced bone destruction [52]. These treatments include chemotherapy, radiotherapy, targeted therapy, and antiresorptive therapy. Aminobisphosphonates and the anti‐RANKL monoclonal antibody denosumab have been approved by the United States Food and Drug Administration for the prevention and treatment of skeletal‐related events in patients with BoMet [237, 238]. Additionally, the bone‐targeted radioactive isotope radium‐223 (^223^Ra) is approved for managing symptomatic metastatic CRPC and painful BoMet in men [239]. In contemporary clinical practice, novel targeted therapies and immunotherapies are increasingly being incorporated. It is essential to tailor treatment strategies according to the individual characteristics of each patient, incorporating factors such as physical condition, tumor molecular subtype, prior treatment history, bone pain severity, and psychological factors.

### 6.1 Surgery

Surgical interventions can preserve limb function and mobility by preventing impending pathological fractures, stabilizing established pathological fractures, managing spinal cord compression, and alleviating pain. In patients with spinal metastases secondary to NSCLC, surgical intervention is associated with significant pain relief and neurological improvement and, in some cases, restoration of ambulatory capacity. Furthermore, a correlation exists between the duration of neurological deficits and survival among patients with NSCLC with spinal cord compression. This observation implies that early intervention may confer a more favorable prognosis [8]. However, owing to its significant physical and functional burden and its inability to prevent the recurrence of BoMet or the development of secondary metastases, surgery is not recommended as a primary treatment option.

### 6.2 Radiotherapy

Radiotherapy has proven effective in alleviating BoMet‐induced pain, preventing pathological fractures, and reducing the requirement for further surgical intervention. It is commonly employed to manage cancer‐induced bone pain across various malignancies, demonstrating an overall pain response rate of approximately 70%, with complete pain resolution achieved in approximately 30% of patients [240].

Radiotherapy modalities for BoMet include external beam radiotherapy (EBRT) and radionuclide therapy. Patients with previously unirradiated BoMet typically achieve significant pain relief and consequent improvement in quality of life following EBRT [241]. For example, in HCC patients with BoMet, EBRT yielded overall pain improvement in 73–99.5% of cases, with complete pain resolution achieved in 17–44% of patients, consequently reducing the requirement for high‐dose analgesic medication [23]. In asymptomatic BoMet patients, prophylactic EBRT can reduce the future risk of pain and skeletal‐related events, although it has not been shown to improve OS [8]. In recent years, stereotactic body radiotherapy (SBRT) has demonstrated the ability to deliver a higher biologically effective dose while minimizing toxicity to surrounding healthy tissues. Multiple studies have indicated that patients with BoMet from solid tumors treated with SBRT exhibit a higher rate of complete pain response than those receiving conventional EBRT [242].

Systemic radionuclide therapy represents a valuable treatment alternative for patients with multifocal BoMet that demonstrates increased uptake in ^9^ ^9^ ^m^Tc‐MDP BS. Strontium‐89 (^8^ ^9^Sr) is the most widely utilized radiopharmaceutical for the internal radiotherapy of BoMet [243]. A recent study demonstrated that the administration of ^8^ ^9^Sr alleviates pain in patients with BoMet located at previously irradiated sites, without inducing severe adverse events [244]. ^89^Sr, ^186/188^Re‐HEDP, and ^153^Sm‐EDTMP exhibited similar red marrow dosimetry and showed comparable outcomes in terms of the average depth of thrombocytopenia, as well as the response rate, magnitude, and duration of symptom relief. However, none of these palliative radiopharmaceuticals demonstrated significant improvement in OS [245]. In contrast, ^223^Ra therapy has been shown to significantly prolong OS and improve the quality of life in patients with advanced PCa. Owing to its affinity for mineralized bone tissue, ^223^Ra is more effective when the tumor burden is low and a greater proportion of tumor cells are in close proximity to the bone surface, suggesting that early administration of ^223^Ra may yield superior therapeutic outcomes [169]. However, compared with ^153^Sm‐EDTMP and ^186/188^Re‐HEDP, ^89^Sr and ^223^Ra exhibited lower average pain response rates (50–60%). Patients treated with ^153^Sm‐EDTMP frequently achieve a reduction in analgesic consumption, whereas the primary benefit of ^223^Ra often manifests as a delay in the requirement for analgesic escalation. Consequently, the research focus on ^223^Ra has shifted toward its survival‐prolonging potential rather than its standalone palliative benefits in end‐of‐life care [245]. Notably, radiotherapy can, in rare instances, elicit a systemic “abscopal effect,” where tumors outside the irradiated field undergo regression, an effect thought to be mediated by activation of the immune system [246]. Furthermore, the combination of radiotherapy with molecular‐targeted agents, such as the EGFR inhibitor gefitinib, has demonstrated the potential for achieving long‐term control of BoMet in specific cancer subtypes [247].

However, radiotherapy is associated with a variety of adverse effects. Lymphocytes are among the most radiosensitive cell types, and radiotherapy directly depletes circulating lymphocytes. It can also indirectly impairs bone marrow stem cell function, resulting in substantial lymphocytopenia 1–2 months posttreatment [8]. Approximately 32.8% of patients treated with ^89^Sr radiotherapy experience marked and prolonged hematologic toxicity, which can progress to Grade 3 or 4 thrombocytopenia [245]. Notably, the combination of ^223^Ra with abiraterone/prednisone has been linked to an increased risk of bone fractures [245].

### 6.3 Chemotherapy

The combination of DTX and prednisone was the first regimen approved for treating mCRPC and improves survival [248]. Conventional chemotherapy effectively targets fast‐dividing cancer cells but shows minimal activity against quiescent DTCs in the bone marrow niche [37]. Patients with persistent bone marrow DTCs following DTX treatment exhibit significantly shorter DFS compared with those who convert to a DTC‐negative status. Specific adjuvant chemotherapy regimens have been shown to eliminate CK^+^ tumor cells in the bone marrow in 48.3% of patients, which correlates with a reduced risk of distant metastasis and improved OS. Furthermore, the efficacy of chemotherapy is underscored by its capacity to induce DTC apoptosis. Apoptotic DTCs were detected in 48% of patients receiving primary systemic chemotherapy, and this cohort experienced a lower recurrence risk than patients without evidence of DTC apoptosis [37]. Collectively, these findings demonstrate that eradicating bone marrow DTCs through chemotherapy contributes to reduced metastatic dissemination and prolonged patients survival.

### 6.4 Pharmacotherapy

#### 6.4.1 Bisphosphonates

Bisphosphonates are the first and most extensively utilized bone‐targeting agents. Bisphosphonates demonstrate high binding affinity for hydroxyapatite, the principal inorganic constituent of the bone matrix [249]. Following systemic administration, bisphosphonates undergo rapid clearance from the circulation but selectively accumulate at active bone resorption sites [250]. Bisphosphonates exert anticancer effects on BoMet through three main mechanisms: (1) *Inhibition of osteoclast activity*. Noninvity‐containing bisphosphonates form nonhydrolyzable ATP analogs, inducing osteoclast apoptosis [251]. Nitrogen‐containing bisphosphonates inhibit farnesyl diphosphate synthase (FDPS) in the cholesterol biosynthesis pathway, leading to the accumulation of isopentenyl pyrophosphate and cytotoxic ATP analogs [22]. FDPS inhibition disrupts isoprenylation, impairs Rho GTPases, reduces osteoclast motility and adhesion, and induces apoptosis [252]. (2) *Inhibition of cancer cell activity*. Bisphosphonates not only suppress the progression of local bone disease but also have antitumor effects. Aminobisphosphonates directly affect cancer cells by inhibiting protein isoprenylation, generating proapoptotic ATP analogs, accumulating GTP‐loaded RhoA and cell division control proteins, and interfering with the functions of integrins, AKT, factor‐related apoptosis ligands, and MMPs [253]. Additionally, bisphosphonates inhibit tumor‐induced angiogenesis by reducing VEGF production [254]. Alteration of bone ECM by nitrogen‐containing bisphosphonates also impairs the adhesion capacity of cancer cells [255]. (3) *Enhancement of immune cell activity*. Zoledronic acid reprograms M2 TAMs into M1 phenotypes, suppressing cancer‐associated fibroblast activity and reducing the tumorigenic potential in BRCA and PCa [256]. Aminobisphosphonates activate γδT cells, which target cancer cells that emit danger signals [257]. The use of zoledronate combined with IL‐2 to amplify peripheral γδT cells has improved the prognosis of patients with advanced BRCA or PCa [258].

Alendronate, a second‐generation bisphosphonate, exhibits a high affinity for bone, particularly at sites of active resorption, with a 10–20‐fold greater uptake in metastatic lesions than in normal bone. Ibandronate and zoledronate are third‐generation bisphosphonates; however, clinical trials have demonstrated differences in their efficacies. A Phase III trial in patients with BRCA revealed that zoledronate was superior to ibandronate in preventing SREs (NCT00326820). Recent studies indicated that a 12‐week zoledronate regimen is as effective as a 4‐week schedule for managing BoMet caused by BRCA and other cancers (NCT00320710 and NCT00869206) [259, 260]. In BRCA patients with low‐grade, HR^+^ status, and elevated baseline N‐telopeptide levels, the combination of dasatinib with zoledronate may improve outcomes (NCT00566618) [261]. In CRPC, early zoledronate treatment did not reduce SRE risk (NCT00079001) [262]. However, in NSCLC patients with BoMet, zoledronate has a limited impact on PFS or OS, with a trend toward worse PFS during long‐term follow‐up (NCT00172042) [263]. A Phase II trial showed that compared with everolimus alone, combining everolimus with zoledronate extended the median time to SREs by 4.4 months and PFS by 2.1 months [264]. These findings suggest the need for the targeted use of zoledronate, particularly in combination regimens, for optimal clinical outcomes.

The adverse effects associated with bisphosphonate therapy in the management of BoMet merit careful consideration. A retrospective analysis of lung cancer patients with BoMet receiving bisphosphonates demonstrated that prolonged administration may elevate the risk of adverse events such as renal impairment [265]. Furthermore, osteonecrosis of the jaw and hypocalcemia are also frequently observed adverse effects of bisphosphonate treatment.

#### 6.4.2 Denosumab

Denosumab is a high‐affinity human monoclonal antibody against RANKL that potently inhibits osteoclast formation, function, and survival by blocking its interaction with RANK. In 2020, the National Medical Products Administration approved denosumab for the treatment of SREs in patients with BoMet [266]. Although the effects of zoledronate and denosumab were not significantly different in terms of disease progression or OS in patients with BRCA, denosumab was more effective in delaying or preventing SREs (NCT00321464, NCT00330759) [267, 268]. Denosumab also outperformed zoledronic acid in preventing CRPC‐related SRE (NCT00321620) [269]. Recent research has demonstrated that OPN enters the circulation and migrates to primary tumor sites, where it induces resistance to ICB [11]. Consequently, targeting osteoclasts with denosumab potently enhances the ICB response in extraosseous tumors, whereas bisphosphonate treatment fails to recapitulate this therapeutic benefit [11]. Furthermore, denosumab does not cause nephrotoxicity, making it a safe alternative for patients with renal impairment. Unlike bisphosphonates, which are incorporated into bone minerals, denosumab is not retained in the skeleton, and its suppressive effect on bone resorption is reversible upon treatment cessation. However, Dupont et al. reported the case of a 43‐year‐old patient with lung adenocarcinoma BoMet who sustained four spontaneous rebound vertebral fractures 8 months after discontinuing denosumab [270]. This case highlights the increased risk of rebound osteolysis and fracture following denosumab cessation.

#### 6.4.3 Antiestrogen

Estrogen therapy modulates ER activity and improves OS in patients with BRCA; however, resistance and recurrence remain significant challenges [271]. During BoMet treatment, aromatase inhibitors may induce activating mutations in the ESR1 gene [272]. Clinical studies have shown that combining hormone therapies, such as fulvestrant, with CDK4/6 inhibitors more effectively controls SREs in patients with BoMet than hormone therapy alone (NCT01942135) [273].

#### 6.4.4 Antiandrogen

Several therapeutic options for CRPC, including cytotoxic agents (DTX and cabazitaxel), the immunotherapeutic vaccine sipuleucel‐T, and hormone‐based therapies (enzalutamide and abiraterone), have been shown to prolong OS [274]. Zoledronic acid combined with androgen deprivation therapy at diagnosis prevents SREs for up to 24 months in patients with PCa and BoMet [275].

Enzalutamide, an antiandrogen agent, inhibits the AR by disrupting nuclear translocation, DNA binding, and coactivator recruitment. Compared with placebo, it is approved for patients with CRPC who progress after DTX treatment and significantly extends OS and time to the first SRE (NCT00974311) [276, 277]. However, after an initial sensitivity to enzalutamide, patients often acquire resistance. AR splice variant 7 is a major contributor to resistance [278]. Research has shown that osteoblasts within the BME are indispensable for enzalutamide resistance. Enzalutamide suppresses TGFBR2 expression in osteoblasts. This reduction might involve PTH1R‐mediated endocytosis. Furthermore, PTH1R blockade rescued the enzalutamide‐induced reduction in TGFBR2 levels and improved the response of PCa cells cocultured with osteoblasts to enzalutamide [279].

Another inhibitor of AR signaling is abiraterone, a 17‐α‐hydroxylase/17,20‐lyase inhibitor that effectively blocks testosterone synthesis [280]. Studies using microtissue‐engineered in vitro 3D models have shown that antiandrogen therapy increases cancer cell volume and reduces sphericity in BME, influencing LNCaP cell proliferation and migration [281]. Phase III trials have demonstrated that adding abiraterone to prednisone shortens the time to the first BoMet and provides effective pain relief (NCT00638690) [282]. Abiraterone significantly improved the OS of patients with metastatic CRPC and demonstrated greater efficacy when combined with DTX [283, 284]. A Phase II study revealed that combining ^223^Ra with abiraterone alleviates bone pain and maintains disease stability in most patients with CRPC (NCT02097303) [285].

### 6.5 Clinical Trials

Recent ongoing clinical trials related to the treatment of BoMet tumors are summarized in Table 2, which provides a new perspective for the development of future BoMet research.

**TABLE 2:** Recent clinical trials related to BoMet.

NCT number	Stage	Study type/phases	Objectives	Results	References
NCT02164019	Active, not recruiting	Interventional/–	Evaluate the differences in quality of life and function following long‐stem cemented hemiarthroplasty or intramedullary nailing in patients with BoMet	—	[286]
NCT05115331	Active, not recruiting	Interventional/Phase III	Evaluate the efficacy of hypofractionated (8 Gy in a single fraction) and dose‐escalated palliative (16 Gy in two fractions) radiotherapy in BoMet	—	[287]
NCT04592887	Completed	Interventional/–	Assess the feasibility of FLASH radiotherapy for the palliative treatment of painful BoMet	Ultra‐high‐dose‐rate proton FLASH radiotherapy is clinically feasible, with treatment efficacy and adverse event profiles comparable to those of standard radiotherapy.	[288]
NCT05524064	Active, not recruiting	Interventional/–	Evaluate the toxicity and pain relief provided by FLASH radiotherapy in subjects with painful thoracic vertebral metastases, compared with conventional radiotherapy	—	[289]
NCT05101824	Active, not recruiting	Interventional/Phase II	Explore the efficacy and toxicity of stereotactic ablative radiotherapy (SABR) in the treatment of osseous oligometastases in daily clinical practice	The 1‐year local control rate of SABR is as high as 93.1%, and the incidence of adverse reactions within 1 year is acceptable.	[290]
NCT05038124	Active, not recruiting	Interventional/–	Evaluate the safety and efficacy of preoperative SABR for BoMet	—	[291]
NCT03795207	Active, not recruiting	Interventional/Phase II	SBRT with or without durvalumab (MEDI4736) in oligometastatic recurrent hormone‐sensitive patients with PCa	—	[292]
NCT03143322	Active, not recruiting	Interventional/Phase III	Compare with patients receiving standard treatment only to assess the impact of SBRT on PFS in patients with oligometastases	SABR is a safe and effective treatment method for oligometastases.	[293]
NCT03230734	Active, not recruiting	Interventional/Phase II	Determine the effect of the treatment sequence of ^223^Ra and docetaxel on efficacy in CRPC patients with BoMet	—	[294]
NCT03317392	Active, not recruiting	Interventional/Phase I/II	Clarify the safety and efficacy of the combined treatment of olaparib and ^223^Ra for BoMet in CRPC	—	[295]
NCT03523351	Completed	Interventional/Phase I/II	Compare the differences in therapeutic efficacy between early upfront palliative radiotherapy and standard treatment in patients with BoMet	Prophylactic radiotherapy can reduce skeletal‐related events and improve OS.	[296]
NCT02839291	Active, not recruiting	Interventional/–	Compare the differences in therapeutic efficacy between intravenous and oral antibone‐resorption drugs	—	[297]
NCT05280067	Active, not recruiting	Interventional/Phase II	Explore the feasibility of using Zetame for treating bone defects resulting from metastatic tumors in the spinal vertebrae via percutaneous implantation	—	[298]
NCT02051218	Active, not recruiting	Interventional/Phase III	Compare the efficacy of denosumab when administered at a dose of 120 mg every 12 or 4 weeks	—	[299]
NCT01833806	Completed	Interventional/Phase IV	Clarify the treatment efficacy of ExAblate for pain caused by BoMet	After ExAblate treatment, the number of patients experiencing pain relief is significantly higher than that of patients with pain progression.	[300]
NCT02609828	Completed	Interventional/Phase III	Evaluate the efficacy and safety of tanezumab in subjects with cancer pain due to BoMet who are receiving background opioid therapy	Tanezumab shows potential in alleviating pain caused by BoMet. However, the durability of Tanezumab remains to be further confirmed, and the risk of intra‐articular pathologic fractures cannot be excluded.	[301]

## 7 Novel Delivery System and Novel Agents

Although there are currently various treatment options for BoMet, none are effective. This may be due to the dense structure of bone, which leads to incomplete drug delivery or marked heterogeneity among patients. Therefore, it is essential to focus on novel drug delivery systems and to develop new treatment methods targeting BoMet.

### 7.1 Novel Delivery System

The unique composition and vascular nature of bone tissue create substantial obstacles for targeted drug delivery and the effective accumulation of antitumor agents in metastatic lesions. Although high‐dose, prolonged bisphosphonates can achieve therapeutic concentrations, this strategy risks compromising the therapeutic index and leads to severe systemic toxicity. These limitations underscore the critical need for developing novel and efficient drug delivery systems.

#### 7.1.1 Doxorubicin@DBMs–ALN

Owing to its simple chemical structure and high bone affinity, alendronate is an ideal targeting ligand that can be chemically conjugated to other functional moieties to construct efficient drug delivery systems. Dextran is a biodegradable, biocompatible polysaccharide. The abundant hydroxyl groups enable diverse chemical modifications, making it a versatile material for drug delivery systems [302]. Ye et al. designed a redox‐responsive amphiphilic copolymer, 1,2‐distearoyl‐sn‐glycero‐3‐phosphoethanolamine (DSPE)–dextran–ALN, by conjugating (1) alendronate as both a bone‐targeting ligand and antiresorptive agent, (2) dextran as the hydrophilic segment, and (3) DSPE as the hydrophobic moiety. This copolymer was subsequently loaded with doxorubicin (DOX) to form DOX@DBMs–ALN NPs [303]. In vitro studies demonstrated the efficient cellular uptake of DOX@DBMs–ALN by A549 lung cancer cells, with successful nuclear delivery of DOX, resulting in potent cytotoxicity. The in vivo results revealed specific accumulation of DOX in BoMet lesions. Furthermore, DOX@DBMs–ALN not only suppressed tumor growth but also significantly reduced osteolytic lesions with minimal systemic toxicity [303].

#### 7.1.2 Trastuzumab–ALN

BME promotes the release of HER2^+^ CTCs, facilitating the formation of secondary metastases in patients with HR^+^ BRCA [38]. Although HER2‐targeting antibodies such as trastuzumab (Tras) and pertuzumab are standard treatments, their efficacy against BoMet remains limited [304]. To address this issue, Tian et al. developed an innovative bone‐targeting strategy using pClick conjugation to site‐specifically link ALN to Tras [304]. In xenograft mouse models, the resulting Tras–ALN conjugate significantly enhanced antibody accumulation within BoMet niches. This targeted delivery specifically eliminated bone marrow macrophages, suppressed tumor growth in the bone, and reduced secondary metastasis to other organs [304]. This platform for bone‐specific antibody delivery represents a promising strategy to enhance the efficacy of antibody therapies against BoMet.

#### 7.1.3 BP@Gel–CD[SA] Hydrogel

Although immunotherapy is currently the first‐line treatment for advanced lung cancer, its efficacy against BoMet remains limited [22]. This is due to the “cold” immune microenvironment in BoMet, resulting in reduced responsiveness to ICB. An injectable hydrogel‐based platform (BP@Gel–CD[SA]) was engineered for photothermal immunotherapy by incorporating a STING agonist and black phosphorus nanosheets (BPNSs) within a cyclodextrin‐modified matrix [305]. Photothermal therapy converts optical energy into localized heat, inducing direct apoptosis of tumor cell at elevated temperatures. This process facilitates immune cell infiltration into the TME, thereby enhancing the immunogenicity of “cold” tumors. Concurrently, STING agonists encapsulated within the nanocarriers stimulate IFN‐I production. This initiates a signaling cascade that activates innate and adaptive immune cells, eliciting potent antitumor responses. As efficient photothermal converters, BPNSs degrade into inorganic phosphates that serve as fundamental building blocks for bone repair. These phosphate components act as osteogenic mineralization agents, actively promoting bone formation [305]. In vivo and in vitro experiments also confirmed that BP@Gel–CD[SA] can not only prevents tumor recurrence and activates systemic antitumor immune responses but also promotes new bone formation at tumor‐induced bone destruction sites, improving bone strength in the affected areas [305].

#### 7.1.4 MgFe_2_O_4_@ZOL

As stated previously, hyperthermia exerts a direct cytotoxic effect on cancer cells. Microwave ablation (MWA) induces coagulative necrosis via microwave energy within an electromagnetic field. MWA offers therapeutic benefits, including minimal side effects, effective alleviation of cancer‐related pain, and enhanced chemotherapeutic drug internalization [306]. However, MWA risks damaging adjacent normal tissues. Advances in nanotechnology led to the creation of numerous microwave‐responsive nanomaterials aimed at safeguarding normal tissues while improving tumor treatment efficacy. Shu et al. developed MgFe_2_O_4_@ZOL nanocomposites that release Fe^3^^+^, Mg^2^^+^, and zoledronate specifically within the acidic TME [306]. The released Fe^3^^+^ depletes intracellular glutathione and catalyzes H_2_O_2_ to generate •OH, thereby inducing chemodynamic therapy (CDT). Furthermore, Mg^2+^ and zoledronate promote osteoblast differentiation. Consequently, these MgFe_2_O_4_@ZOL NPs enable the targeted and selective heating of tumor tissue, augmenting microwave thermal therapy [306]. Both in vitro and in vivo studies have demonstrated that the synergistic effects of targeted delivery, glutathione depletion‐enhanced CDT, and selective MTT significantly contribute to antitumor efficacy and bone regeneration [306].

#### 7.1.5 PB@LC

Chen et al. developed a biomimetic nanosystem featuring a crosslinked peptide–lipoic acid micelle platform coated with a fused cell membrane derived from bone marrow MSCs and PCa cells. This system achieved homotypic targeting of PCa and bone‐homing capabilities by precisely targeting the BoMet niche. While DTX is the first‐line chemotherapy for BoMet CRPC, its efficacy is limited by drug resistance and adverse reactions. Sterol regulatory element‐binding proteins (SREBPs) serve as key enzymes in lipid metabolism [307]. Interference with SREBP expression directly affects PCa progression and metastasis by inhibiting PI3K/AKT signaling, enhancing DTX sensitivity, and eliminating resistance in PCa. Using PB@LC, Chen et al. delivered DTX and si‐SREBP1 to the BoMet niche in a mouse model of BoMet CRPC and achieved excellent therapeutic outcomes [308].

#### 7.1.6 NP‐αCD137 Ab‐F4/80

In vivo experiments have demonstrated that liposomal clodronate‐mediated depletion of monocytes/macrophages significantly suppresses BRCA BoMet. Besides, CD137 expression on macrophages promotes their migration and differentiation into osteoclasts [134]. Jiang et al. designed and synthesized a novel liposomal NP targeting F4/80, encapsulating an anti‐CD137 antibody (NP‐αCD137 Ab‐F4/80). Following the establishment of a mouse BoMet model, NP‐αCD137 Ab‐F4/80 was intravenously administered three times at a low dose of 6 µg antibody per 20 g of body weight. Compared with control groups (PBS, αCD137 Ab, or NP‐αCD137 Ab), NP‐αCD137 Ab‐F4/80 significantly inhibited osteoclast differentiation and BRCA BoMet. Moreover, treatment with NP‐αCD137 Ab‐F4/80 did not significantly alter the percentage of splenic monocytes/macrophages, the infiltration and activation of T‐cells in tumor tissue, or body weight compared with other groups. Furthermore, combination therapy with NP‐αCD137 Ab‐F4/80 and the Fra1 inhibitor SKLB816 demonstrated superior therapeutic efficacy against BoMet compared with PBS, NP‐αCD137 Ab‐F4/80 alone, or SKLB816 alone [134].

### 7.2 Novel Agents

#### 7.2.1 Fingolimod Hydrochloride (FTY720)

FTY720, a sphingosine analog and potent S1P receptor modulator, is phosphorylated by sphingosine kinases, primarily SK2, and binds to S1PR1, 3, 4, and 5. FTY720 induces the internalization of S1P1, thereby inhibiting S1P activity. Recent studies have shown that the addition of exogenous S1P and FTY720 has no significant effect on RANKL‐induced osteoclastogenesis in marrow‐derived macrophages. However, FTY720 inhibits osteoclastogenesis induced by VitD3 alone [155]. S1P and SPHK1 independently enhance the chemotaxis of osteoblasts and T‐cells, whereas FTY720 abolishes these effects. Additionally, FTY720 suppressed S1P‐ and VitD3‐stimulated RANKL expression in osteoblasts. In cocultures of marrow‐derived macrophages and osteoblasts, FTY720 significantly reduces osteoclastogenesis (Table 3).

**TABLE 3:** Novel agents for cancer BoMet.

Name	Target	Tumor	Molecular mechanism	References
FTY720	S1PR	—	Inhibit osteoclastogenesis	[155]
cephalomannine	UBE2S	PCa	Inhibit tumor cell proliferation, migration and invasion; reduction osteoclast numbers	[309]
BW‐755C	5‐Lipoxygenase; COX	BRCA	Inhibit tumor cell proliferation and migration	[138]
S6	SOST	BRCA	Inhibit STAT3 phosphorylation; remodeled the BME	[310]
LTMA16D5	SCUBE2	BRCA	Inhibit SHH release; inhibits osteoblast differentiation	[175]
Sonidegib	Hedgehog signaling	BRCA	inhibits osteoblast differentiation; increase NK cell infiltration	[175]
5G12	Siglec‐15	BRCA	Enhance antitumor immunity; inhibits osteoblast differentiation and secondary dissemination	[311]
αIL‐1β	IL‐1β	BRCA	Enhance antitumor immunity	[121]
p38i	p38 MAPK	BRCA	Inhibit tumor cell migration and secondary dissemination	[116]
ATI‐450	MK2	BRCA	Inhibit tumor cell migration and secondary dissemination	[116]
DHA	CCL2/CCR	Lung cancer	impair macrophage recruitment and M2 polarization	[312]
LTMA1G11	IL‐20RB	Lung cancer	Inhibit tumor cell proliferation, migration and invasion	[125]
Verteporfin	YAP–TEAD	HCC	Inhibit osteoclastogenesis	[213]

#### 7.2.2 Cephalomannine

In PCa cells, UBE2S expression progressively decreases with increasing concentrations of cephalomannine [309]. Furthermore, cephalomannine inhibits tumor cell proliferation in a dose‐dependent manner and blocks G1/S phase progression, invasion, and migration of PCa cells. In a BoMet model established via tail artery injection in nude mice, cephalomannine treatment reduced bone destruction and metastatic lesion counts in a dose‐dependent manner compared with controls. Cephalomannine treatment significantly prolonged the time to the event endpoint. TRAP/ALP double staining revealed a significant reduction in the number of osteoclasts in the treatment group, indicating the suppression of bone destruction. Notably, cephalomannine showed no significant toxicity to the heart, liver, or kidneys of nude mice [309].

#### 7.2.3 BW‐755C

BW‐755C, a dual inhibitor of the 5‐lipoxygenase and COX pathways, significantly reduces BRCA cell growth. In addition, BW‐755C treatment eliminates the ability of osteoclastic lipids and AA to promote cancer cell migration [138].

#### 7.2.4 S6

Elevated expression of SOST is associated with the development of BoMet and poor prognosis in patients with BRCA. Mechanistically, the SOST–STAT3 interaction amplifies TGF‐β/KRAS signaling, thereby promoting tumor growth and BoMet. Computational screening identified a candidate compound, S6, which disrupts the SOST–STAT3 interaction, inhibits downstream STAT3 phosphorylation, and impairs the growth of BRCA organoids [310]. Furthermore, S6 administration reduced the incidence of BoMet and favorably remodeled BME by promoting differentiation of bone marrow MSCs and suppressing osteoclast activity.

#### 7.2.5 SCUBE2 Neutralizing Antibody (LTMA16D5) and Sonidegib

As mentioned above, SCUBE2 expression is elevated in ER^+^ BRCA cells, facilitating the release of SHH from tumor cells. This activates the Hedgehog signaling pathway in MSCs, thereby promoting osteoblast differentiation, suppressing NK cell activity, and ultimately enhancing the colonization of BRCA cells within BME [175]. Wu et al. developed a SCUBE2‐neutralizing antibody targeting the CUB domain, which is essential for SHH cleavage, and identified the IgG monoclonal antibody LTMA16D5. LTMA16D5 was shown to inhibit SHH release from BRCA cells in a dose‐dependent manner, resulting in the suppression of Hedgehog pathway activation and osteoblast differentiation of MSCs [175]. In the established left ventricular injection mouse model, daily intraperitoneal administration of LTMA16D5 markedly reduced the burden of BoMet. This reduction was accompanied by a decreased in osteoblast numbers and an increase in NK cell infiltration at metastatic sites. Sonidegib, a pharmacological inhibitor of the Hedgehog signaling pathway, was found to lower ALP expression in peri‐metastatic bone regions and restore NK cell infiltration in bone lesions. As a result, it alleviated the bone metastatic burden and prolonged survival in the mouse model. In the case of spontaneous orthotopic BRCA, sonidegib did not affect primary tumor growth but did significantly suppress BoMet [175]. Collectively, these findings suggest that targeting SCUBE2 in BRCA cells and the Hedgehog signaling pathway in MSCs represents an effective therapeutic strategy for the prevention and treatment of BoMet. However, whether the combined use of LTMA16D5 and sonidegib yields superior antimetastatic efficacy warrants further investigation.

#### 7.2.6 Siglec‐15 Blocking Antibody (5G12)

Aberrant hypersialylation of tumor surface glycans enables cancer cells to evade immune surveillance by engaging inhibitory Siglec receptors on leukocytes. Siglec‐15, a member of the Siglec protein family, is normally expressed in a subset of myeloid cells but is markedly upregulated in human cancer cells and tumor‐infiltrating myeloid/macrophage populations [313]. Beyond its role as an immune checkpoint, elevated Siglec‐15 expression within the BME significantly enhanced tumor‐induced osteoclastogenesis. Moreover, differentiated osteoclasts in this niche suppress T‐cell function through Siglec‐15‐mediated interactions. In an intratibial mouse model, treatment with the anti‐Siglec‐15 antibody, 5G12, showed no apparent signs of toxicity and produced striking therapeutic efficacy, with most mice achieving complete tumor regression after the second administration. By day 18, only 11% of the control mice survived, whereas all the mice in the 5G12‐treated group remained alive [314]. Treatment with 5G12 also markedly reduced the number of osteoclast within the BoMet niche and prevented tumor‐induced bone destruction. Notably, when BRCA cells were injected into the right hind limb of mice, bioluminescence imaging revealed that as the bone lesions progressed, metastatic dissemination extended to major organs, particularly the lungs and liver. However, the incidence of secondary metastases was markedly reduced following 5G12 treatment, indicating that blocking of Siglec‐15 effectively suppressed secondary dissemination initiated by bone lesions [311].

#### 7.2.7 αIL‐1β Neutralizing Antibody

Recent studies have revealed that spontaneous BoMet of BRCA in mice exhibits an increased infiltration of granulocytes accompanied by elevated T cell populations, the majority of which differentiate into immunosuppressive Tregs. Granulocyte‐derived IL‐1β was identified as a key driver in maintaining the immunosuppressive BME [121]. Mice were treated with neutralizing antibodies against IL‐1β (αIL‐1β) or the T‐cell immune checkpoint receptors PD‐1/TIGIT, either individually or in combination (αIL‐1β + αPD‐1 or αIL‐1β + αTIGIT). Compared with mice receiving isotype control antibodies, all treatment groups showed a markedly reduced BoMet burden. Compared with αIL‐1β alone, immune checkpoint inhibition conferred greater protection against bone destruction [121]. Notably, although αPD‐1 therapy appeared to reduce the incidence of BoMet, approximately one‐third of mice developed lethal cytokine storm responses, resulting in no significant improvement in OS. In contrast, αPD‐1 and αIL‐1β + αTIGIT combination treatments were most effective in reducing severe BoMet lesions, with the αIL‐1β + αTIGIT combination producing the most pronounced decrease in mortality. The αIL‐1β + αTIGIT combination significantly increased the ratio of CD8^+^ cytotoxic T cells to CD4^+^ helper T cells. Furthermore, in vitro coculture assays demonstrated that blocking the TIGIT–CD155 signaling axis between granulocytes and cytotoxic T cells alleviated T‐cell dysfunction. These findings suggest that dual neutralization of IL‐1β and TIGIT constitutes an effective therapeutic strategy to mitigate BRCA BoMet by restoring and reinforcing antitumor immune responses.

#### 7.2.8 p38MAPK Inhibitor (p38i) and p38MAPK–MK2 Inhibitor (ATI‐450)

The antitumor effect of p38i is cell nonautonomous, as luminal‐B BRCA cells are not directly sensitive to it. Instead, p38i suppresses the secretion of protumorigenic factors from stromal cells. Since MK2, a downstream effector of p38 MAPK, stabilizes the mRNAs of protumor cytokines like IL‐6, we also evaluated the p38 MAPK–MK2 inhibitor ATI‐450 [116]. In mouse models, p38i reduced BoMet to a degree similar to that of PTX. The antimetastatic effect of p38i was systemic, resulting in a significant reduction in visceral metastases comparable with PTX. ATI‐450 effectively inhibited both bone and visceral metastases. Furthermore, combining MK2Pi with PTX significantly prolonged mouse survival compared with treatment with either agent alone. Importantly, both p38i and ATI‐450 treatments preserved bone density as effectively as zoledronic acid, while simultaneously reducing tumor burden [116]. Therefore, targeting the p38 MAPK/MK2 pathway represents a promising strategy for achieving dual antitumor and bone‐protective effects in BRCA.

#### 7.2.9 Dihydroartemisinin

Dihydroartemisinin (DHA) is a first‐generation artemisinin derivative and a well‐established antimalarial agent [314]. Emerging evidence has demonstrated its potent anticancer properties both in vitro and in vivo, including the inhibition of tumor cell proliferation/migration, induction of apoptosis, and suppression of angiogenesis across multiple cancer types [312]. Mechanistic studies have revealed that DHA reprograms macrophage polarization by promoting the M1 phenotype and suppressing M2 differentiation, thereby attenuating lung cancer cell invasion and migration [312]. In vivo, DHA treatment modulates the M1/M2 balance in both primary lung tumors and bone metastatic niches, resulting in significant suppression of tumor growth and BoMet. Furthermore, DHA disrupts the CCL2/CCR2 axis, impairing macrophage recruitment and accumulation [312]. These findings suggest that DHA as a promising therapeutic candidate for the simultaneous targeting of primary lung cancer and BoMet via TME immunomodulation.

#### 7.2.10 IL‐20RB Antibody (LTMA1G11)

The tumor‐specific overexpression of IL‐20RB in lung carcinomas versus its limited expression in normal tissues underscores its potential as a therapeutic target. He et al. have developed LTMA1G11, a neutralizing monoclonal antibody targeting the extracellular domain of IL‐20RB [125]. This antibody specifically detects IL‐20RB in lung cancer cell lysates and critically blocks the interaction between IL‐19 and IL‐20RB. In vitro studies demonstrate that LTMA1G11 suppresses macrophage‐induced activation of the JAK1–STAT3 pathway in lung cancer cells and inhibits organoid formation [125]. In vivo experiments involved inoculating lung cancer cells into the left ventricle of nude mice, followed by LTMA1G11 treatment 1 week later (each animal received intraperitoneal injections of 100 µg LTMA1G11 or IgG every other day). Upon completion of the treatment cycle, the tumor burden in BoMet lesions was significantly reduced in the LTMA1G11 group, and the proliferation of intratumoral cells was significantly suppressed [125]. Furthermore, healthy mice were administered the same dosage of LTMA1G11 as in the metastasis experiment, but for an extended duration of 4 weeks. Sustained LTMA1G11 treatment showed no significant effects on body weight or blood composition in healthy mice, demonstrating the drug safety profile of the LTMA1G11 antibody [125].

#### 7.2.11 Verteporfin

Zhang et al. demonstrated that HCC cells promote BoMet via the RNF219/α‐catenin/LGALS3 axis, wherein yes‐associated protein 1 (YAP1)/transcriptional enhancer factor domain family members (TEAD) interaction critically regulates LGALS3 transcription (Table 3) [213]. The YAP–TEAD antagonist verteporfin suppressed LGALS3 expression and impaired osteoclast differentiation. In a prevention mouse model, coadministration of verteporfin following intracardiac inoculation of highly metastatic HCC cells significantly reduced TRAP^+^ osteoclasts on bone surfaces compared with vehicle controls. In a therapeutic intervention model initiated after established BoMet, verteporfin treatment suppressed metastatic burden, diminished osteoclast presence at the tumor‐bone interface, and prolonged survival [213].

## 8 Preclinical Models and Translational Gaps

To advance the investigation of tumor BoMet mechanisms and the development of novel therapies, a variety of preclinical models have emerged and undergone rapid development in recent years, ranging from traditional 2D cell cultures to more physiologically relevant 3D systems and various murine models. Understanding the strengths and weaknesses of each model and employing them in rational combinations is essential for bridging the gap between fundamental research and clinical translation.

### 8.1 In Vitro Model

**FIGURE 6:**
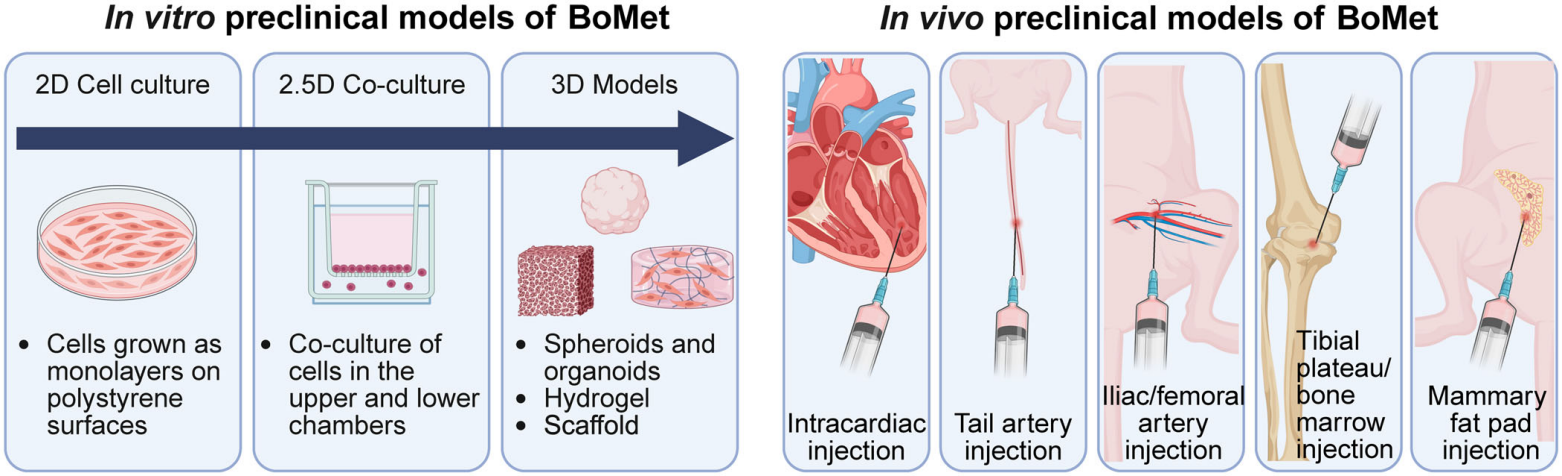
Preclinical models of cancer bone metastasis. In vitro preclinical models have evolved from simple 2D monolayers and transwell‐based 2.5D cocultures to more physiologically relevant systems employing scaffolds, hydrogels, and patient‐derived organoids that better recapitulate the human tumor microenvironment. In vivo models encompass direct tibial plateau or bone marrow injections, routes modeling hematogenous dissemination (intracardiac, tail artery, iliac/femoral artery), and orthotopic implantation that most faithfully recapitulates the complete metastatic cascade. Created with BioRender.com.

Owing to its simplicity and cost‐effectiveness, 2D monolayer culture on polystyrene plates remains the gold standard for in vitro cancer modeling (Figure 6). This approach enables highly reproducible investigations under tightly controlled conditions, typically generating data faster than in vivo models, making it ideal for high‐throughput drug screening. In BoMet research, 2D cultures are commonly used in culturing tumor cells, osteocytes, osteoclasts, osteoblasts, and other cell types. These models are then employed to assess various cellular activities, differentiation status, tumor cell proliferation, invasion, and metastatic potential. However, 2D models poorly replicate the BoMet niche due to the absence of key biophysical, biochemical, and biomechanical cues, which limits their utility in accurate therapeutic screening and mechanistic investigations of BoMet [315]. The transwell system is commonly employed for coculture experiments, wherein two or more cell types are cultured in separate compartments that share the same culture medium. Typically, cancer cells are seeded in the upper chamber, whereas BME cells and tissues are placed in the lower compartments. This setup provides a robust platform for investigating cancer cell dissemination, with a particular focus on cell migration and invasion driven by chemotaxis or haptotaxis [315].

Organoids are generated in vitro from stem cells or organ‐specific progenitor cells via a self‐organizing developmental processes. Compared with other tumor models, organoid cultures more faithfully reproduce the characteristics of the original tumor. The matrices commonly used for organoid cultures resemble the native TME, thereby enhancing the physiological relevance of the model. Dhimolea et al. cultured spheroids derived from HR^+^ BRCA and PCa cells, along with patient‐derived organoids, within a 3D ECM [316]. These cultures were maintained both alone and in coculture with bone marrow stroma, revealing that tumor cells rely on HR signaling for anchorage‐independent growth. These findings provide crucial insights into the mechanisms underlying hormone therapy resistance and elucidate the dynamic interplay between cancer cells and their microenvironment during metastasis [316]. While organoids offer more physiologically relevant 3D models for BoMet research, creating organoid systems that accurately reflect the heterogeneity of patient tumors and their associated microenvironments continues to be a significant challenge.

Over the past decade, biofabrication technologies have advanced substantially, enabling the engineering of increasingly sophisticated in vitro 3D tissue models of bone, vasculature, and various TME. Tumor spheroids represent one of the earliest 3D culture paradigms, formed through the self‐assembly of cells into 3D aggregates in suspension [317]. Tumor spheroids are widely used in cancer research because of their compatibility with straightforward and well‐established techniques. Larger spheroids develop gradients of oxygen and nutrients, resulting in corresponding gradients of proliferation and regional heterogeneity. This heterogeneity establishes distinct zones of proliferation, quiescence, and necrosis, alongside a hypoxic core that closely mimics the pathophysiology observed in vivo. Consequently, the gene and protein expression profiles of tumor spheroids resemble those of in vivo tumors in key aspects, such as proliferation, metabolism, angiogenic potential, and drug resistance, more closely than those seen in 2D culture models [317]. Certain in vitro bone matrix models consist of over 95% mineralized bone tissue, including embedded osteocytes, and are enriched with specific bone proteins and minerals, thus closely replicating the highly mineralized nature of native BME. For instance, human osteoblast‐derived tissue‐engineered constructs can be indirectly cocultured with patient‐derived PCa xenografts to assess paracrine signaling and molecular interactions within a patient‐mimicking microenvironment [315]. This engineered biomimetic microenvironment, characterized by a high degree of mineralization and embedded osteocytes, enables PCa xenografts derived from the lymph nodes and BoMet of primary PCa to exhibit strong osteomimicry at the transcriptomic, proteomic, and mineralization levels [318]. The 3D‐printed scaffolds have emerged as superior experimental models for recapitulating complex multicellular physiological events under controlled conditions. These systems enable effective investigation across physical, cellular, and molecular dimensions, particularly for assessing cellular chemosensitivity to therapeutic agents. Using GelMA–nHA bioprinted scaffolds to coculture osteoblasts with MDA‐MB‐231 BRCA cells, Zhou et al. demonstrated that osteoblasts promoted VEGF expression in cancer cells, whereas cancer cells suppressed osteogenic activity [316]. Kar et al. reported enhanced proliferation rates of BRCA cells cultured within polycaprolactone‐hydroxyapatite‐clay (PCL/HAPClay) scaffolds compared with conventional 2D PCL/HAPClay films [316].

### 8.2 Animal Models

Given the limitations of in vitro models in recapitulating the complex interactions between tumors and BME, in vivo animal models are indispensable for elucidating the mechanisms underlying BoMet. Well‐established animal models are essential for identifying therapeutic targets of BoMet and evaluating the efficacy of novel drug candidates (Figure 6).

The most widely used animal model for BoMet is established by the intracardiac injection of cancer cells into mice, which enables the development of multifocal metastases (e.g., in the tibia, femur, humerus, and vertebrae). Cell lines with a heightened propensity for BoMet can be derived through repeated cycles of harvesting tumor cells from BoMet foci and reinjecting them via the intracardiac route [319]. Researchers have also employed tail artery injections to achieve high‐volume delivery of cancer cells specifically to the hind limb bone marrow [320]. Despite being practical and minimally invasive, both techniques carry a significant risk of systemic tumor dissemination, often resulting in premature animal death due to metastasis to other organs. The iliac or femoral artery allows for localized delivery of cancer cells to the hind limb skeleton while minimizing dissemination to other organs. This approach is particularly valuable for investigating subsequent metastatic spread from the bone [36, 321]. The main limitation is the technical complexity of these procedures, which require microsurgical equipment. To minimize dissemination to other organs and simplify the procedure, tumor cells may be injected directly into the tibial plateau or bone marrow cavity. However, this invasive approach can cause local bone damage, and such models only mimic the colonization and expansion stages of BoMet [120]. The orthotopic model, which uses spontaneous bone‐metastasizing cell lines injected into the mammary fat pad, overcomes this limitation by recapitulating the full metastatic cascade. Additionally, this model's compatibility with immunocompetent mice allows for the examination of cancer–immune interactions during BoMet [120]. Although these animal models are widely used, they often fail to fully recapitulate human disease. Humanized models address this limitation by providing a more physiologically relevant human BME, thereby enabling a better simulation of patient tumor progression. One approach involves implanting femoral head tissue from hip replacement patients into immunodeficient mice, followed by orthotopic injection of cancer cells into the bone graft. This model supports the spontaneous metastasis of injected cancer cells within the human bone niche. It not only facilitates the investigation of BoMet but also bridges basic research and clinical translation, aiding in the identification of novel therapeutic targets [322]. However, this model has not been widely adopted, likely due to its technical complexity and high cost.

The translational failure of most drugs, from animal models to clinical trials, stems from interspecies differences in physiology (e.g., immunity, gene expression, and homeostasis). Consequently, there is a critical lack of mouse models that are both fully representative and conveniently operable to accurately recapitulate the entire stage of human BoMet.

## 9 Conclusion and Prospects

BoMet is a serious complication of advanced cancer that compromises patients’ quality of life and OS. Current detection methods often fail to identify early‐stage BoMets, leading to diagnoses predominantly after symptom onset. Consequently, substantial research efforts have been made to identify biomarkers capable of predicting BoMet development, enabling risk stratification for intensified follow‐up and early interventions. However, the predictive capacity of these biomarkers has not been fully characterized. Although some candidate biomarkers show promise in preclinical testing, others have demonstrated limited utility in clinical settings. Robust validation through large‐scale clinical trials is essential to determine which biomarkers possess sufficient predictive power for clinical translation. Liquid biopsy‐compatible biomarkers offer distinct advantages over tissue biopsies, as they enable serial sampling and longitudinal risk assessment. The dynamic nature of liquid biopsies holds significant potential for monitoring disease progression and therapeutic responses, thereby facilitating personalized management of patients with BoMet. Colonization and expansion of tumor cells within the bone can be detected through imaging, indicating the presence of BoMet in patients with cancer. However, various imaging modalities require applications tailored to specific clinical scenarios.

There is a growing research focus on the molecular mechanisms of BoMet, which has significantly advanced our comprehension of its extensive regulatory networks. Although substantial progress has been made in elucidating the complex mechanisms underlying BoMet, certain processes, particularly those governing secondary metastasis from established bone lesions, require deeper investigation. Furthermore, a more comprehensive exploration of the interactions among diverse cellular subsets within the BoMet niche is warranted. Such efforts will provide a more holistic understanding of the sequential events that drive cancer dissemination to bone.

Future research should concentrate on enhancing the resolution of single‐cell sequencing to better decipher the cellular composition and dynamics of the TME. Spatial transcriptomics now enables the visualization and quantification of transcriptomes at single‐cell resolution within their native tissue architecture. These integrative approaches provide a more precise understanding of the molecular mechanisms underlying BoMet. Integration with spatial metabolomics further clarifies region‐specific metabolic reprogramming within BoMet, providing compelling evidence for the metabolite‐driven mechanisms behind tumor dissemination to the bone. A deeper understanding of the molecular mechanisms of BoMet is likely to aid in identifying novel and promising therapeutic targets. Ongoing innovations in sequencing technologies will support the clinical implementation of precision medicine for BoMet.

Although the current management of BoMet involves multimodal approaches (surgery, radiotherapy, chemotherapy, and pharmacological agents), the overall therapeutic efficacy remains suboptimal. Consequently, future research should prioritize the prevention and treatment of BoMet, guided by several key objectives: (1) Delineating the complex mechanisms of BoMet to provide a robust foundation for targeted and effective therapies; (2) identifying biomarkers with high sensitivity and specificity to facilitate early diagnosis, risk assessment, and treatment stratification; and (3) discovering novel therapeutic targets to develop more effective and personalized treatment regimens. Our fundamental understanding of disease biology is expanding rapidly. As these discoveries transition from basic science to preclinical and clinical applications, the prognosis of patients with BoMet is expected to improve substantially.

## Author Contributions

JW and BL designed and wrote the manuscript. DY and YY drafted and revised the manuscript. ZH and SW participated in drawing figures and organizing tables. All the authors have read and agreed to the published version of the manuscript.

## Funding

This work was supported by the National Natural Science Foundation of China (Grants 82403240, 82372834, 82173129, and 82203809) and Tongji Hospital (Huazhong University of Science and Technology) and Foundation for Excellent Young Scientist (24‐2KYC13057‐15).

## Ethics Statement

The authors have nothing to report.

## Conflicts of Interest

The authors declare no conflicts of interest.

## Data Availability

Data availability is not applicable to this review as no new data were created or analyzed in this study.
